# Dynamic interchange of local residue–residue interactions in the largely extended single alpha-helix in Drebrin

**DOI:** 10.1042/BCJ20253036

**Published:** 2025-04-23

**Authors:** Soma Varga, Bálint Ferenc Péterfia, Dániel Dudola, Viktor Farkas, Cy M. Jeffries, Perttu Permi, Zoltán Gáspári

**Affiliations:** 1 Pázmány Péter Catholic University Faculty of Information Technology and Bionics, 1083 Budapest, Práter u. 50/A, Budapest, Hungary; 2 HUN-REN - ELTE Protein Modeling Research Group, ELTE Eötvös Loránd University, Pázmány Péter Sétány 1/A, Budapest, Hungary; 3 European Molecular Biology Laboratory, Hamburg Unit, c/o Deutsches Elektronen-Synchrotron, Notkestraße 85, 22607, Hamburg, Germany; 4 Department of Biological and Environmental Science, Nanoscience Center, University of Jyväskylä, P.O. Box 35, Jyväskylä, FI 40014, Finland; 5 Department of Chemistry, Nanoscience Center, University of Jyväskylä, P.O. Box 35, Jyväskylä, FI 40014, Finland; 6 University of Helsinki, Helsinki Life-Science Institute – Institute of Biotechnology, P.O. Box 56, Helsinki, FI 00014, Finland

**Keywords:** ensemble modeling, molecular dynamics, NMR spectroscopy, SAH, SAXS

## Abstract

Single alpha-helices (SAHs) are protein regions with unique mechanical properties, forming long, stable, monomeric helical structures in solution. To date, only a few naturally occurring SAH regions have been extensively characterized, primarily from myosins, leaving the structural and dynamic variability of SAH regions largely unexplored. Drebrin (developmentally regulated brain protein) contains a predicted SAH segment with unique sequence characteristics, including aromatic residues within the SAH region and a preference for arginine over lysine in its C-terminal half. Using circular dichroism (CD) and NMR spectroscopy, combined with small-angle X-ray scattering (SAXS) measurements, we demonstrate that the Drebrin-SAH is helical and monomeric in solution. NMR resonance assignment required specific 4D techniques to resolve severe signal overlap resulting from the low complexity and largely helical conformation of the sequence. To further characterize its structure, we generated a structural ensemble consistent with Cα, Cβ chemical shifts and SAXS data, revealing a primarily extended structure with non-uniform helicity. Our results suggest that dynamic rearrangement of salt bridges and potential transient cation-π interactions contribute to the formation and stabilization of both helical and non-helical local conformational states.

## Introduction

Single alpha-helical (SAH) domains form stable helices in solution and are proposed to possess unique mechanical properties such as acting as constant force springs, lever arms, and spacers between protein regions with different functions. The first SAH domains were found in caldesmon [1] and in myosin 10 [2], prompting the introduction of the term SAH. To date, very few atomic-level structural studies are available on SAH domains, and most of these focus on the SAH in myosin VI, one of the first SAH regions discovered. Structures available in the PDB include the X-ray structure of myosin VIIa (5WST [3]) SAH and the recently described DNA-binding ‘KER’ region of the chromatin assembly factor Cac1 (8DEI [4]), as well as the solution NMR structure of the myosin VI SAH (6OBI [5]). All three are straight helical structures with no direct representation of the actual dynamic internal motions characteristic to SAH regions.

In contrast, a number of dynamic features have been proposed to contribute to SAH stability and its mechanical properties, including the constant reorganization of ionic interactions [6]. Using the NMR chemical shift and residual dipolar coupling data available from myosin VI [5], we have previously generated a structural ensemble to explore the nature and extent of the internal motions in the myosin VI SAH region, as part of the demonstration of our ensemble-generation pipeline DIPEND [7]. Our ensemble suggests the presence of kinking motions with largely retained overall helicity. The exact extent of helix breaks has been proposed to represent a major difference between SAHs and less stable helical peptides, with SAHs exhibiting generally more stable straight conformations with higher end-to-end distances [8,9].

**Figure 1:**
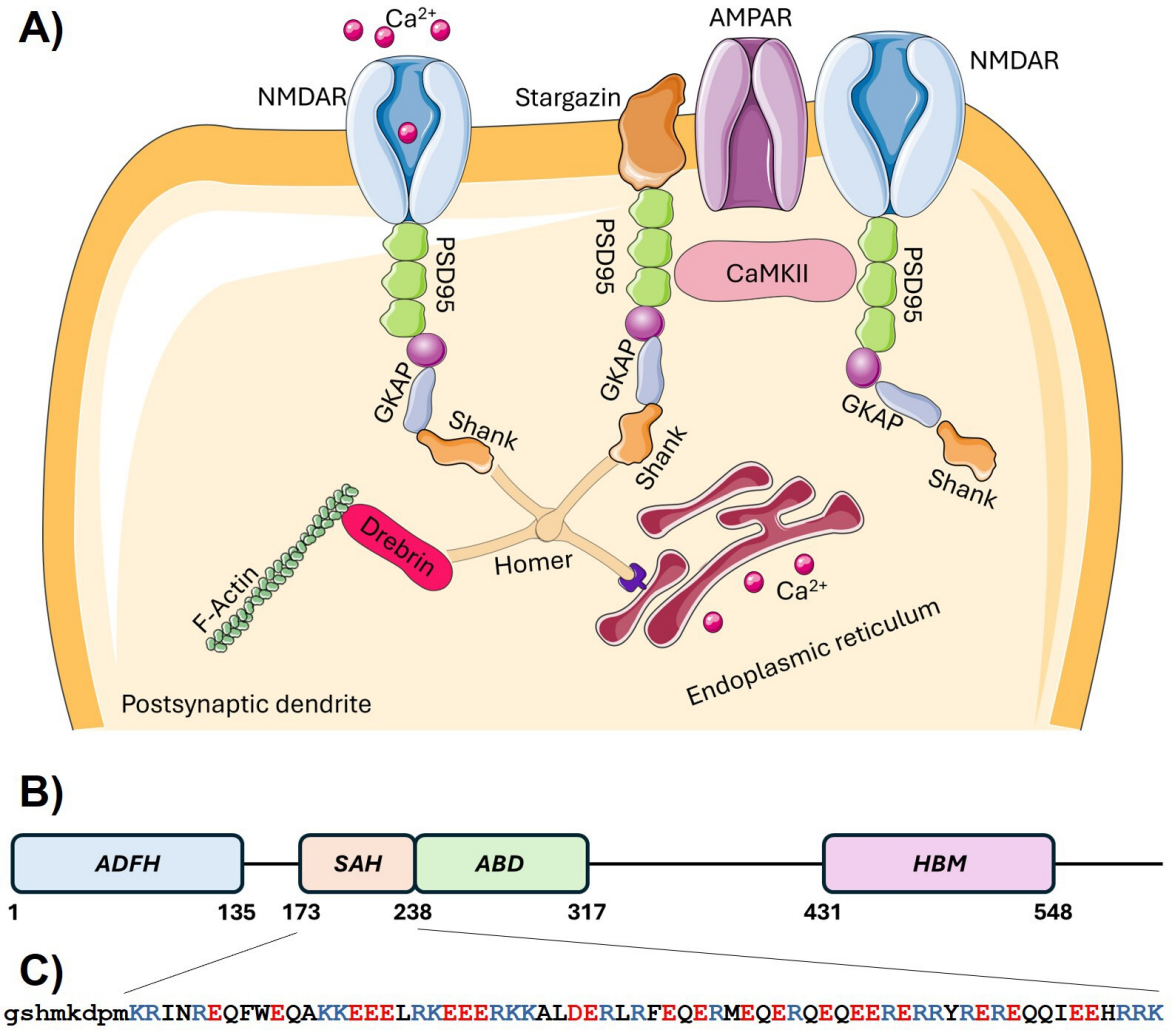
Cellular localization and structural organization of Drebrin. **(A**) Localization and role of Drebrin in the organization of the postsynaptic density. Drebrin links the scaffold protein Homer to the actin cytoskeleton. (**B**) Schematic depiction of Drebrin domains and regions. (**C**) Sequence of the Drebrin-SAH construct investigated in this study. Amino acids in lowercase letters at the N-terminus belong to the expression tag. In the SAH region, positively charged residues are shown in blue, and negatively charged in red. ABD, actin-binding domain; ADF-H, actin-depolymerizing factor homology domain; HBM, Homer-binding motif; SAH: single alpha-helix**.**

The protein Drebrin (developmentally regulated brain protein) is involved in linking postsynaptic complexes to the actin cytoskeleton (Figure 1A). It contains an N-terminal actin depolymerization factor homology (ADF-H) domain and a number of non-globular regions, some of which have been proposed to participate in actin binding (Figure 1B). A segment following the ADF-H domain and adjacent to the actin-binding domain was previously predicted to form an SAH domain [10,11] (Figure 1C). This segment has also been predicted to form a coiled coil, but the protein was shown to be monomeric [12,13]. The putative SAH region of Drebrin is practically fully conserved in a number of orthologs (Supplementary Table S10), and Drebrin exhibits several characteristic differences relative to the most well-studied myosin SAHs, most notably the presence of a neighboring Phe-Trp residue pair near its N-terminus, a Phe in the central, as well as Tyr and a His in the C-terminal region of the SAH. The myosin VI SAH contains no aromatic residues, whereas the myosin VIIa SAH construct has a single Tyr in the N-terminal and two His residues in the central region and near the C-terminus. In addition, the distribution of Arg and Lys residues is characteristically different. The Drebrin-SAH exhibits a biased distribution with practically no lysines in its C-terminal half except the last residue of the predicted region, again in contrast with the myosin SAHs where no such marked bias can be observed, although the N-terminal segment of the myosin VIIa SAH [14], bearing the Tyr residue, is also devoid of Lys residues (Supplementary Figure S1; Supplementary Table S1). It has been proposed that the Lys and Arg amino acids have slightly different contributions to SAH helicity and stability [6]; thus, sequence variation in different natural SAH regions might contribute to their biological versatility. We believe that the investigation of multiple natural SAH regions can reveal functionally relevant features of this structural element. Therefore, we reckoned that experimental characterization of the Drebrin-SAH segment extends our general knowledge on SAH structure and dynamics. In this study, we used NMR and small-angle X-ray scattering (SAXS) data and integrated these into an ensemble-based atomic-level structural model.

## Results and discussion

### Circular dichroism confirms high level of helicity of the Drebrin-SAH region

**Figure 2:**
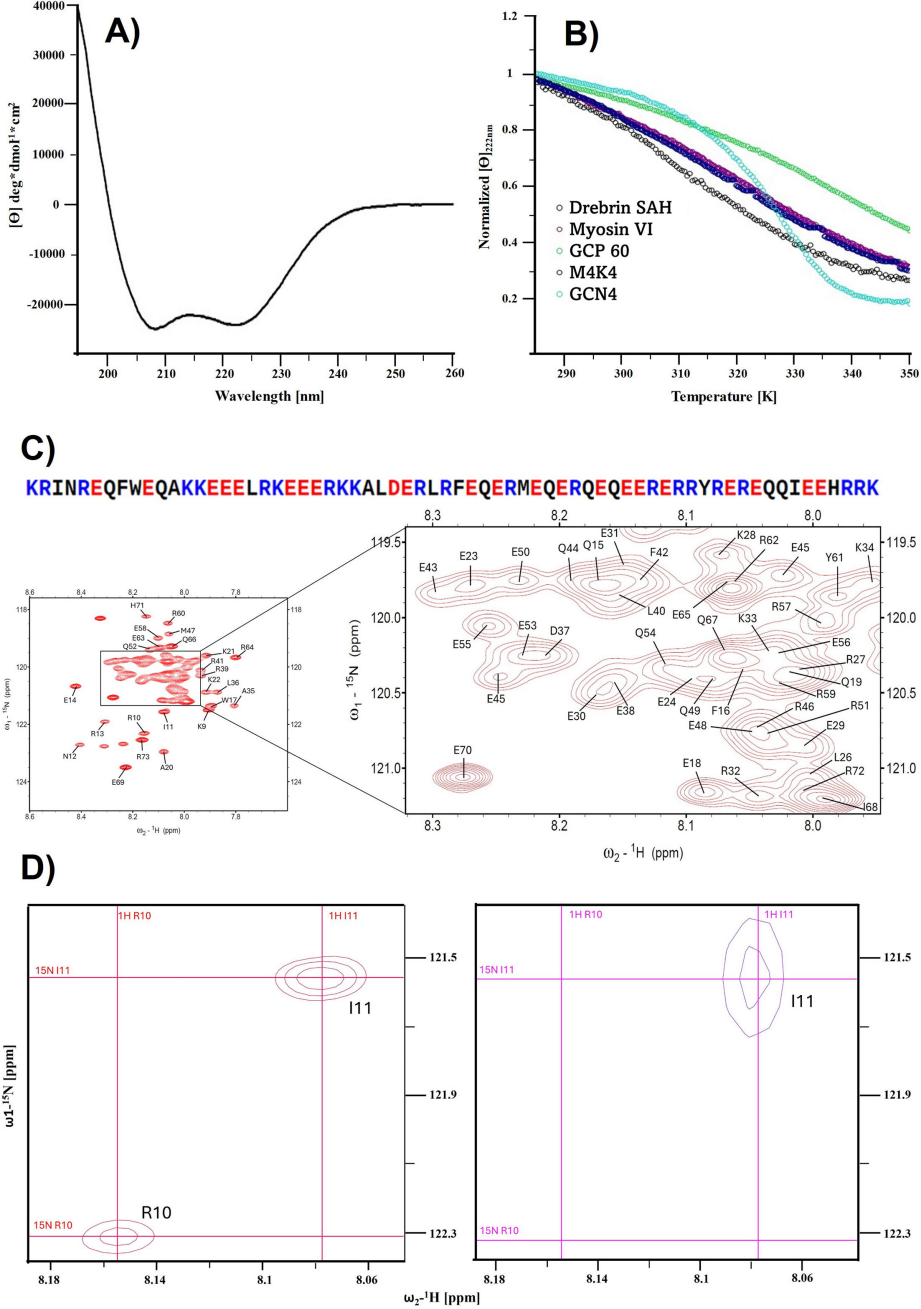
CD and NMR spectroscopy results of the Drebrin-SAH motif. **(A**) Curve fitting to the experimental CD data of the Drebrin-SAH shows characteristic peaks at 215 and 222 nm indicating alpha-helical structure. (**B**) Temperature denaturation: Mean residual ellipticity at 222 nm normalized to the value measured at 285 K, as a function of temperature (gray circles). Comparison with results obtained for the GCN4 coiled-coil, (cyan), as well as the single alpha-helix (SAH) regions of MYO6 (purple), M4K4 (dark blue) and GCP60 (green). (**C**) Sequence of the Drebrin-SAH domain. The low complexity and the high amount of charged residues in the protein sequence is highlighted with blue (positive charge) and red (negative charge). The panel displays the ^1^H-^15^N HSQC spectrum of the Drebrin-SAH in 50 mM NaCl, 17 mM NaH_2_PO_4_, 3 mM Na_2_HPO_4_, pH 6.0 at 25°C. The expansion highlights severely overlapping NH resonances in the central region. The assignments of chemical shifts are shown with one-letter amino acid codes. (**D**) Sequential assignment by walking through the NH signals correlated by the 4D intraresidual i(HCa)N(Ca)CONH experiment. Left,^1^H-^15^N HSQC spectrum of the Drebrin-SAH in 50 mM NaCl, 17 mM NaH_2_PO_4_, 3 mM Na_2_HPO_4_, pH = 6.0 at 25°C showing the position of two following residues. Right, 4D i(HCa)N(Ca)CONH view of the same spectral width, starting from R10. The complete backbone resonance assignment was obtained with the combinative use of 3D HNCO, iHNCO, HCCCONH, and the 4D i(HCa)N(Ca)CONH experiments using the following strategy: first, COi and COi-1 peaks were picked in iHNCO and HNCO spectra, respectively. Then the corresponding NH peaks were accurately picked on the HSQC plane, which otherwise would have been impossible due to the extreme overlap. With these in hand, we could use the 4D i(HCa)N(Ca)CONH spectrum with dimension order ^1^H,^15^N,^15^N,^13^C. Assuming the NH and CO peaks are picked correctly, a 4D cross-peak observed in the previously described way unambiguously led us to the NH peak of the following residue. The CO correlations were further proven with the HNCO experiments, which in itself was found to be insufficient to correlate with the iHNCO peaks, due to its high degeneracy. CD, circular dichroism.

Circular dichroism (CD) spectra recorded from the Drebrin-SAH demonstrates that the recombinant protein forms a stable alpha-helix in solution (Figure 2A). The Drebrin-SAH is estimated to exhibit about 63% helicity, slightly lower than that of the GCP60 and M4K4 SAH regions reported earlier [15] (Table 1). However, this value is strongly influenced by the fact that in our 74-residue Drebrin-SAH construct, the first eight residues originate from the non-native linker sequence that remains after tobacco etch virus (TEV) cleavage that probably has no regular secondary structure. Throughout this article, we provide amino acid numbering for the full human Drebrin (UniProt Q16643) in square brackets following the residue positions in our construct. Temperature dependence of Drebrin-SAH was monitored at 222 nm (Figure 2B) and compared with previously reported SAH segments from myosin VI, GCP60, and M4K4 in addition to the coiled-coil region of GCN4. While the GCN4 coiled coil exhibits a clear sigmoidal curve indicating cooperative thermal unfolding with a characteristic melting point, the curve recorded for the Drebrin-SAH shows a continuous decrease in the measured helicity at higher temperatures, consistent with the behavior of previously investigated SAH segments of M4K4, GCP60, and myosin VI. These data are consistent with a non-co-operative unfolding of the Drebrin-SAH region, indicating that it does not form a coiled coil but rather form a stable monomeric helical structure in solution.

**Table 1:** Helicity values calculated from the CD data of the previously published GCP60 and M4K4 SAH regions, as well as the Drebrin-SAH.

	GCP60	M4K4	Drebrin-SAH
Residue nr	56	53	74
MRW [g]	134.97	133.36	131.46
C [uM]	19.85	7.64	15
MW [g]	7558.32	7068.08	9728.04
MRE [deg*dmol^-1^*cm^2^]	-27506.11	-28754.40	-24904.01
Helicity [%]	67.7	79.2	63.4

CD, circular dichroism. SAH, single alpha-helix.

### Secondary chemical shifts are consistent with helical structure

The high abundance of charged residue repeats and the low complexity of the Drebrin-SAH protein sequence provided an extremely challenging task to perform resonance assignment, as the conventional triple resonance spectra were burdened with severe signal overlap (Figure 2C). The putative single-alpha helical structure carries in itself the parallel alignment of the amide vectors along the transverse axis of the helix, resulting in a remarkably low dispersion of the amide NH peaks in the ^1^H-^15^N HSQC spectra. Notably, similar spectral crowding was observed for the myosin VIIa SAH [14] domain. Sequential walkthrough was only feasible with the 4D i(HCa)N(Ca)CONH experiment, which yielded in CO(i)-N(i)-N(i + 1)-HN(i + 1) correlations, making it possible to distinguish between overlapping signals in the ^15^N and ^1^H dimensions [16] (Figure 2D). The (H)CC(CO)NH and H(CC)(CO)NH experiments also produced some ambiguity, but starting from anchor residues, such as I11 [175 in the full UniProt entry Q16643], W17 [181], L26 [190], A35 [199], M47 [211], T61 [225], and I68 [232], was sufficient to unambiguously identify all backbone and most of the side chain connections. All experiments used for resonance assignment are listed in Supplementary Table S2. The list of assigned chemical shifts has been deposited to BMRB with the ID:52729. Secondary Cα and Cβ chemical shifts, calculated using POTENCI [17], provided further proof of the helical structure of Drebrin-SAH (Supplementary Figure S2). The highest helicity appears in the middle of the SAH region, and it decreases slightly toward the N-terminal and C-terminal parts, similar to the trends reported for the myosin VI SAH [5].

### SAXS indicates that Drebrin-SAH prefers extended structural states in solution

The SAXS data and primary data analysis of Drebrin-SAH measured at three sample concentrations are summarized in Supplementary Figure S3 and Supplementary Table S3. In general, the SAXS profiles (Supplementary Figure S3A) appear similar, where all three profiles exhibit a relatively featureless monotonic decay in the scattering intensities. Indeed, when the SAXS datasets in Supplementary Figure S3A are scaled to protein concentration in mg/ml, and then compared, all three profiles are statistically similar (*χ*^2^ = 1.07–1.09; CorMap-P = 0.01–0.02), indicating no discernible concentration-dependent effects on the scattering intensities. The radius of gyration (*R_g_*) is consistent across all three samples, spanning 2.9–3.2 nm, as it is the concentration-independent molecular weight (MW) estimate of 8.5 kDa derived from scattering invariants (Supplementary Figure S3D and Supplementary Table S3). The MW estimate from SAXS was additionally validated using size exclusion chromatography-multi-angle laser light scattering (SEC-MALLS)/refractive index (RI) measurements from the same Drebrin-SAH sample, yielding an MW average of 11 kDa (Supplementary Table S3; Supplementary Figure S5). Combined, the MW results demonstrate that the Drebrin-SAH is monomeric under the sample conditions used for SAXS, which are identical to those sample conditions used for NMR. This has been further validated as the protein mass from the MS experiment was calculated to be 9727.0 Da, with 0.75 Da difference from the expected 9727.75 Da. The difference can be attributed to the accuracy of the MALLS-SEC method, which is one magnitude less than the Q-TOF MS; however, the oligomeric state could still be reliably assessed. Both methods clearly indicate that the region is monomeric under the conditions used. Of note, the *R_g_* of the Drebrin-SAH monomer is comparatively large for a ~10-kDa protein. For example, the *R_g_* of bovine serum albumin (BSA) – a much larger 66-kDa globular protein (SASBDB ID: SASDFQ8) – is 2.8 nm, which is smaller than the *R_g_* of the Drebrin-SAH. This simple ‘*R_g_*-to-MW’ comparison suggests that the mass distribution of Drebrin-SAH is not evenly distributed in all directions around the center of mass but is rather distributed toward structurally extended mass/volume states.

The subsequent dimensionless Kratky plots for all three samples (Supplementary Figure S3B) attest to the extended structural-state hypothesis, where a systematic increase in the transformed intensities is observed as *sR_g_* increases. The dimensionless Kratky plots are absent in the typical ‘bell-shaped maximum’ of a globular protein or a plateau at increasing *sR_g_* as is often observed for intrinsically disordered proteins [18]. All the corresponding *p(r*) profiles (Supplementary Figure S3C) yield a highly anisotropic distribution of real-space scattering-pair distances that extend out to a maximum particle dimension, *D*_max_, of 12–13.5 nm, which is, once again, considerable for a 10-kDa protein (e.g., compared with BSA *D*_max_ = 8 nm). However, the *p(r*) profiles, although highly anisotropic, do not conform to what would be expected for cross-sectionally thin and stiff-rod-type particles that are characterized by a sharp increase in the frequency of scattering-pair distances toward a definable maximum in *p(r*) at low *r* (relating to the rod cross-section) followed by a systematic near-linear decay in *p(r*) as *r* approaches *D*_max_ [19]. For example, the *p(r*) profile calculated from a single-conformation, extended alpha-helix model of Drebrin-SAH (Supplementary Figure S3C, inset) retains ‘stiff-rod-like’ features in the distance distribution that are otherwise less-evident in the *p(r*) determined from the SAXS data. This suggests that although the Drebrin-SAH prefers to adopt extended states, there may be components of flexibility in the structure that deviate the Drebrin-SAH structure away from a truly stiff-rod-like conformation.

An analysis of the decay in the scattering intensities in the mid-s region of the scattering data (~0.55–2.0 nm^-1^) was performed to evaluate the mass-fractal scaling relationships of the Drebrin-SAH samples. In the larger angle region of the scattering profile, the relationship between *I(s*) and *s* may be generalized as:


$$I(s)\propto s^{-Dm}$$

where *Dm* is the mass-fractal dimension of the particles [20]. The *Dm* encodes information relating to the interatomic distances on different length scales internal to a molecule, in effect describing the scaling relationship between the mass enclosed by the particle volume and its linear dimensions (shape characteristics). This parameter is determined from the negative slope of the decay in the scattering intensities in the intermediate-angle region of the scattering profile when the data are plotted as Log*I(s*) vs. Log(*s*) (Supplementary Figure S4A). The *Dm* is, in turn, related to the Flory exponent, *υ* (where *υ* = 1/ *Dm*), that describes the relationship between the *R_g_* of a polymer and the number of monomer units – in this case amino acids – comprising the chain length [18,21]. For globular/compact particles/solids, the *Dm* ranges from 3 to 4 (*υ* ≈ 0.25–0.33); for a flat planar ‘disc’-like object, *Dm* is 2 (*υ* ≈ 0.5); for a self-crossing random-walk chain, the *Dm* is also 2 (*υ* ≈ 0.5), while for a well-solvated swollen chain or intrinsically disordered protein, the *Dm* is around 1.7–1.8 (*υ* ≈ 0.55–0.59); and for stiff highly extended rod-like particles, the *Dm* is even lower, approaching ~1 *(υ* ≈ 1). For the Drebrin-SAH samples, the *Dm* is ~1.36 (*υ* = 0.73–0.74; Supplementary Figure S4A), which is a too-shallow decay in the scattering intensity for the scaling relationship to be categorized as highly flexible/disordered, and too large to be considered as a purely stiff and rod-like. What the *Dm* analysis suggests, when combined with the *R_g_* observations, dimensionless Kratky plot, *p(r*), and *D*_max_ descriptions, is that the Drebrin-SAH is present in solution as a set of structures that have a likely preference toward sampling extended conformations and that there is an allowance for limited bending/flexibility in these states, as opposed to adopting a well-defined single stiff-rod-like extended structure. Subsequent *ab initio* bead-modeling and the spatial alignment of the globally restored shape derived from SAXS with the conformational ensemble of alpha-helical variants determined from NMR (Supplementary Figure S4B) are entirely consistent with the extended, multi-state conformational sampling of Drebrin-SAH in solution. The SAXS results have been deposited to SASDB with the ID SASDVV6.

### Structural ensembles of the Drebrin-SAH region reveal an extended yet dynamic helix

**Figure 3:**
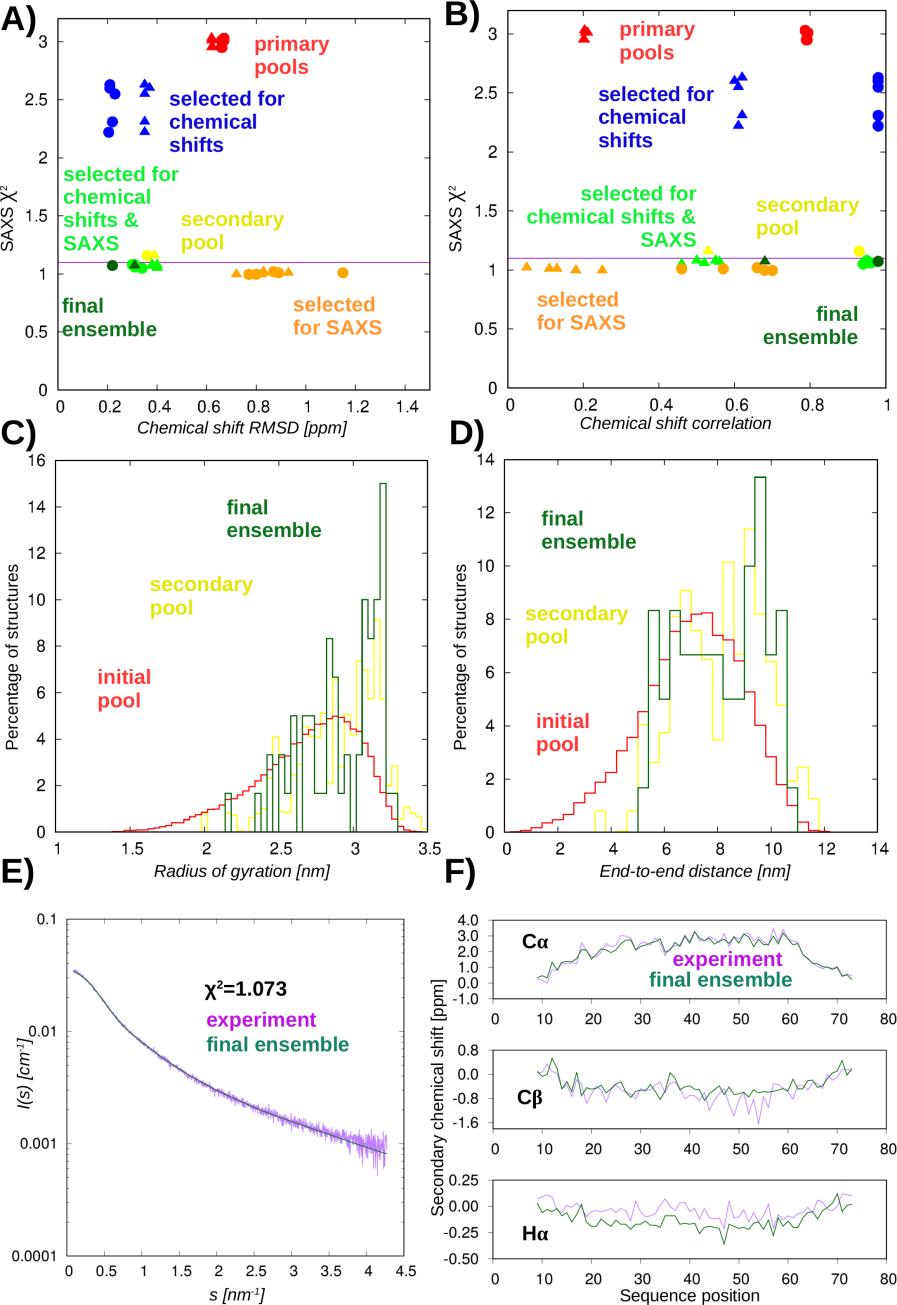
Correspondence of the different ensembles to experimental data. **(A**) and (**B**) Correspondence to SAXS and chemical shift data using RMSD and correlation for secondary chemical shifts (RMSD is the same for primary and secondary shifts). Filled circles represent Cα and filled triangles Cβ chemical shifts. (**C**) Distribution of the radius of gyration and (**D**) end-to-end distance in conformers of the initial pool, the secondary pool, and the final ensemble. (**E**) Comparison of the experimental SAXS curve with the one back-calculated for the final ensemble. (**F**) Comparison of experimental secondary chemical shifts with those estimated for the final ensemble. The lines connecting the points are shown for better visualization of the trends.

We have generated an initial pool of 30,000 structures with regionally adjusted, high helicity (Supplementary Figure S6) using DIPEND [7] and GROMACS [22] (vide infra Computational methods). After randomly choosing 5000 structures, we have selected subsets from these based on their correspondence to Cα/Cβ chemical shifts and the measured SAXS curve. After repeating these steps five times, we generated a secondary pool from the models included in any of the selections and subjected each of them to a 20-ns molecular dynamics (MD) run with the backbone coordinates fixed. The final ensemble was selected from this secondary pool for correspondence with the Cα/Cβ chemical shifts and the measured SAXS curve (Supplementary Figure S7). To assess the ensembles generated at each step, additional evaluations and selections were performed (Figure 3A and B).

Acceptable *χ*^2^ values for the SAXS fit could only be obtained for ensembles explicitly selected for correspondence with the SAXS data. Our analysis of the *R_g_* and end-to-end distance reveals that SAXS data favor more extended structures than chemical shifts alone. Selection only for chemical shifts improves the SAXS fit slightly but not beyond the acceptance limit, whereas the selection for only SAXS data renders the correspondence with the chemical shifts even worse than that of the initial pool. This observation justifies the simultaneous use of both data types to obtain conformers that reflect both the local structure governed by the chemical shifts and the overall shape reported by the SAXS measurements. Such ensembles exhibit only slightly worse fitting to these data than the best ones selected for only one of the data types (Figure 3A and B). Hα chemical shifts, not used in any of the selections, behave similarly to Cα/Cβ shifts in terms of improvement, consistent with the fact that they are mostly sensitive to local conformation, similar to Cα shifts [23]. The selections were performed to minimize the RMSD between the experimental and back-calculated chemical shifts. RMSD is independent of the use of primary or secondary chemical shifts. In contrast, the correlation between calculated and experimental secondary shifts is a much more sensitive measure than using primary chemical shifts. The high correlation for sequence-corrected secondary chemical shifts indicated that the trends in local structural preferences are reproduced acceptably.

The *R_g_* of the conformers selected for SAXS data either alone or in combination with chemical shifts is in fairly good agreement with the value of 2.9 nm estimated directly based on the SAXS measurements. End-to-end distance between the Cα atoms of the first and last residues suggests that the construct does not adopt an entirely straight helical structure but still prefers a largely extended conformation (Figure 3C and D).

The final ensemble consists of 60 conformers and exhibits very good correspondence both with the SAXS (Figure 3E) and chemical shift data (Figure 3F); thus, it is expected that the ensemble adequately reproduces both the local and global structural features of the Drebrin-SAH region. Of course, the limited number of conformers is not expected to represent all aspects of the actual structural diversity in the SAH region, but arguably, our selection procedure largely reduces the possibility of overfitting and, hence, provides a conservative ensemble-based representation. Principal component analysis of the selected conformers with respect to the conformational diversity of the initial pool reinforces this conclusion, that is, the different selections sample only well-defined regions represented by the first and third principal components (PCs). The first PC largely describes the compact/extended nature of the molecule, whereas the third PC corresponds to a bending motion (Supplementary Figure S8). Thus, the inclusion of arbitrary non-native conformers that might improve the correspondence to experimental data randomly by simply enhancing the diversity of the ensemble is expected to be negligible.

**Figure 4:**
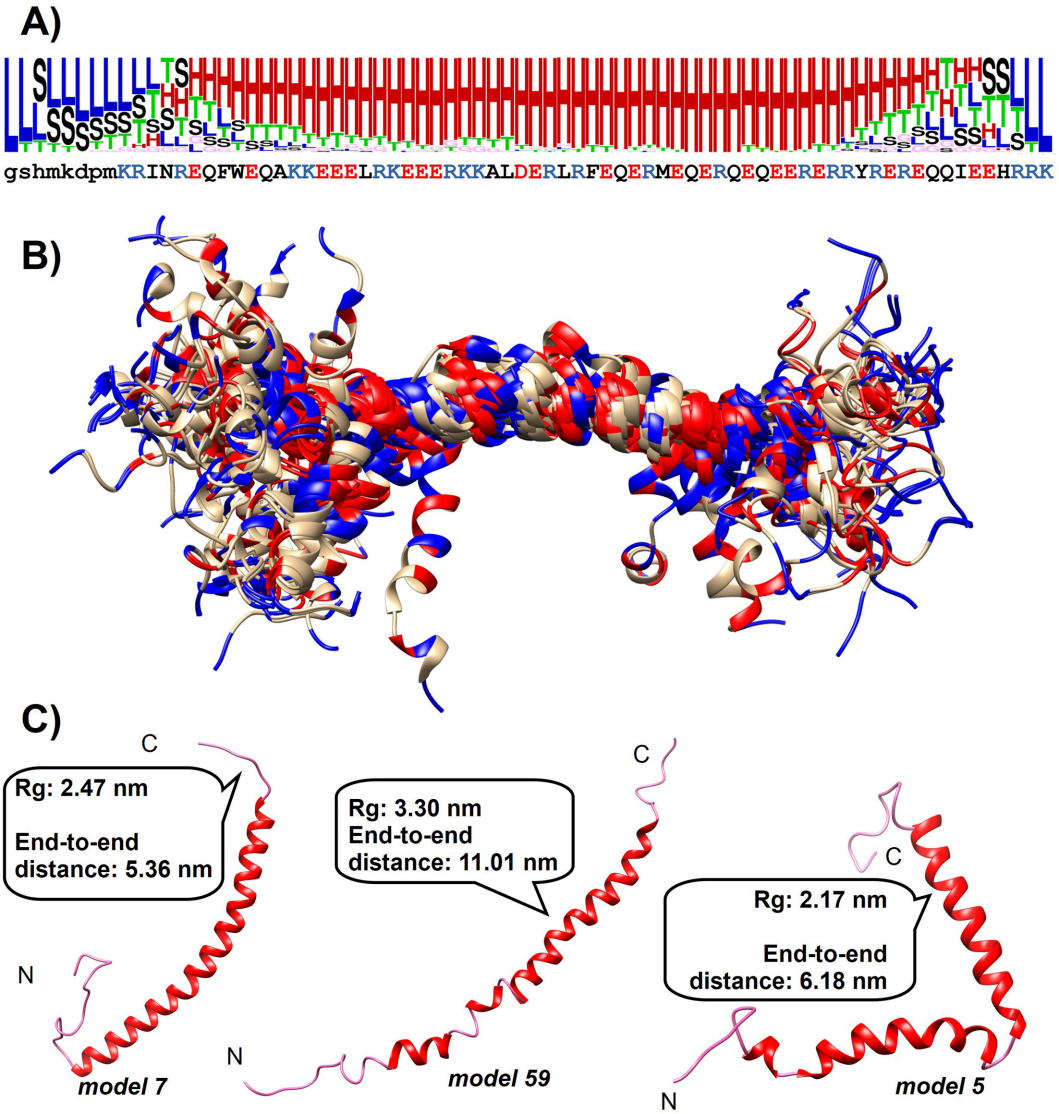
Overall structure of the Drebrin-SAH region. **(A**) Secondary structure logo of the Drebrin-SAH region reflecting the secondary structure state probabilities obtained via DSSPcont. The sequence of the construct is shown below the logo. (**B**) Ribbon representation of the final selected ensemble of 60 conformers for the Drebrin-SAH region. For clarity, the first eight residues, corresponding to the expression tag, are not shown. Conformers have been superimposed over residues 26–59 [190-223] with high helicity. Positively charged residues are colored blue, negatively charged red. (**C**) Conformers of the final ensemble with the lowest end-to-end distance (model 7), lowest radius of gyration (model 5) and the model for which both these are the largest (model 59). SAH, single alpha-helix.

DSSPcont analysis, assigning the probabilities of secondary structure states to each residue, reveals a strong helical propensity of the central region of the helix in the final ensemble. The region between residues 26 and 59 [190-223] can be characterized with a high probability of alpha-helical state, with segments 26–31 [190-195] and 38–59 [202-223] with least 90%, including the region 49–52 [213-216] with an assigned 100% of state ‘H’. The N-terminal part, remaining from the expression tag, is largely disordered. Even when considering this, the helicity is decreasing from the central part toward the terminal regions in an asymmetric manner, with a steeper transition toward the C-terminus (Figure 4A). The structures themselves show two kinds of deviations from a straight helix: the terminal regions are unfolded, whereas in the central regions, there are occasional ‘kinks’ of the helix (Figure 4B and C). The structural ensemble has been deposited to PEDB with the ID PED00524.

### Interactions between charged residues are highly dynamic

**Figure 5:**
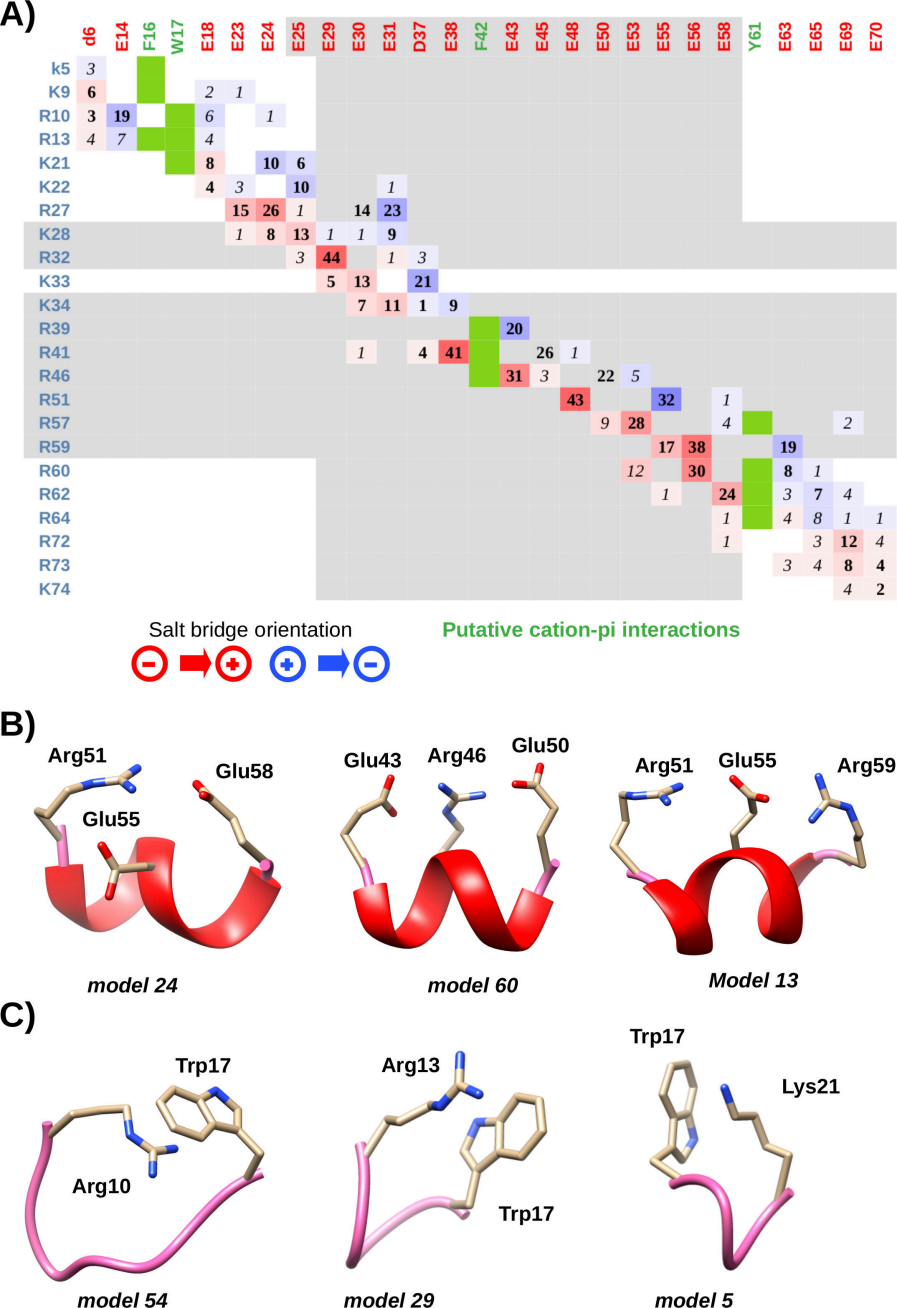
Residue-residue interactions in the Drebrin-SAH region. **(A**) Occurrences of ion pairs between charged residues and putative cation-π interactions in the ensemble. The numbers represent the times a given ion pair was observed. Due to simultaneous ion pair formations, one residue can participate in more than 60 pairs in the ensemble of 60 conformers. Interactions where the Asp/Glu reside is closer to the N-terminus are colored red, opposite orientations are colored blue. i-i+3 and i-i+4 salt bridges are shown with bold numbers, other salt bridges in italics. Residues in segments over 85% helicity according to DSSPcont are shown in gray background. Putative cation-π interactions between aromatic and positively charged residues are shown with green boxes. (**B**) Examples of simultaneous salt bridges in selected conformers. (**C**) Examples of cation-π interactions between Trp17 [181] and positively charged residues in selected conformers. SAH, single alpha-helix.

As expected, being a signature of the SAH structural motif, oppositely charged residues on the same face of the helix can form ionic interactions with each other. The secondary pool, from which the final ensembles were selected, was generated to sample possible side chain conformations. In accordance, the final ensemble reveals diverse interactions between charged residues, even for conformers having virtually identical backbone conformations. However, owing to our selection methodology (vide supra Computational methods), the 60-member ensemble cannot be regarded as reflecting any thermodynamic equilibrium or being large enough to sample all possible interactions that might be relevant. Rather, it can be used to indicate several structural features that might actually occur without being incompatible with the experimental data used. In principle, regarding the full ensemble, almost none of the charged residues forms an interaction solely with one specific, oppositely charged partner (Figure 5A). Hence, each Asp/Glu can establish salt bridges with more than one Lys/Arg and vice versa. The sole exception is Arg39 [203], only forming an ion pair with Glu43 [207], but potentially participating in a cation-π interaction with Phe42 [206] in several conformers. A typical pattern is that residues can interact with partners in both directions, that is, the partner is located either toward the N- or the C-terminus. The most regular pattern, dominated by i-i+3 and i-i+4 salt bridges, is observed for the highly helical region between residues 39 and 59 [203-223] (Figure 5A). Salt bridges between residues at different distances along the sequence (e.g., Glu30 [194]-Arg41 [205] in model 5, shown in Figure 4C) typically indicate non-helical local conformation in the given structures. From the two charged residues located in the expression tag, Lys5 only forms occasional interactions with Asp6, whereas Asp6 can form salt bridges with multiple residues in the SAH region, mostly conforming to the i-i+3 or the i-i+4 pattern.

Closer inspection reveals that there are instances of residues simultaneously interacting with two oppositely charged residues (Figure 5B). There are cases where one residue is ‘sandwiched’ between two oppositely charged partners, one at each side, similar to arrangements described previously based on MD studies [6]. However, for example, Arg51 [215] can make simultaneous interactions with two glutamates located in C-terminal direction along the helix (Figure 5B). Although our 60-membered ensemble does not necessarily sample all possible interactions that occur in the region, this remarkable diversity even on this relatively small sample strongly suggests highly dynamic side chain rearrangements, consistent with earlier suggestions and observations. In our ensemble, the occurrence of salt bridges with the orientation having the negatively charged residue toward the N-terminus is slightly more common than the opposite direction, at odds with the conclusions obtained on model peptides combined with an analysis of PDB structures [24]. The reason of this apparent discrepancy might be that our sampling of conformations and interactions is far from exhausting and also that the previous study focused on Lys→Glu and Glu→Lys pairs in their model peptides, whereas most of the salt bridges in Drebrin-SAH involve Arg residues, especially in the region with the highest helicity, located in the C-terminal half. In our relatively small sample of conformers, lysine side chains seem to behave similarly to those of arginines in terms of being able to form interactions with multiple partners; thus, we observe no hints of different dynamics of salt bridges involving Lys and Arg residues as observed in MD simulations.

### Aromatic–aromatic and cation-π interactions in the Drebrin-SAH region

The most remarkable sequence feature of the Drebrin-SAH is the presence of a Phe-Trp residue pair near its N-terminal part, located within the motif 14EQFWEQ19 [178-183]. The Phe and Trp residues are assigned 60 and 74% ‘H’ state in the final ensemble, respectively; thus, although far from being among the residues with the highest helical character, they are clearly part of the SAH structure. The analysis of the conformations generated reveals that the two aromatic rings can form edge-to-face interactions where one edge of the phenyl ring is within 0.5 nm of the indole ring plane, as can be expected for consecutive aromatic residues in an alpha-helix [25].

From a systematic analysis of the atomic distances between the aromatic rings of Trp, Phe, Tyr residues and the charged groups of Arg and Lys side chains, a number of putative cation-π interactions have been revealed (Figure 5C). Although our conformational ensemble cannot be expected to sample all possible such contacts and cation-π interactions can vary greatly in strength, the estimation of which would require quantum chemical analysis, our observations suggest that basically all aromatic residues can form such interactions with multiple partners. Notably, the indole ring of Trp17 [181] can be close to the amino group of Lys21 [185] or the guanidino groups of Arg10 [174] or Arg13 [177] (Figure 5C). The i-i+4 spacing of Trp17 [181] and Lys21 [185], as well as their aromatic → cationic order, is consistent with a weak stabilizing interaction observed in some helical model peptides [26]. However, this region adopts non-helical conformations in a remarkable number of the selected structures, some of these are still compatible with the presence of the cation-π interaction with one of the preceding arginine residues. In addition, Phe16 [180] can interact with Lys9 [173], or Arg13 [177], and even Lys5 from the remaining linker, the latter two of which can also pair with Trp17 [181] (Supplementary Figure S9). Phe42 [206] can form cation-π interactions with Arg39 [203], Arg41 [205], or Arg46 [210], and Tyr61 [225] with Arg57 [221], Arg60 [224], Arg62 [226], or Arg64 [228]. Regarding residue spacing, the most common pattern observed is the i-i+3 and i-i+4 interactions, compatible with the helical nature of the region. Interestingly, Phe42 [206] is the only aromatics where interaction with the neighboring residue Arg41 [205] might occur. Analysis of the potentially interacting pairs reveals that these do not disturb the helical structure, compatible with the high helicity of both Arg41 [205] and Phe42 [206] (96 and 92%, respectively). In these structures, the orientation of the aromatic plane is parallel with the helix axis rather than the perpendicular one more common in pairs with larger interresidue distances. As in the largely extended SAH region, practically all residues are solvent-exposed, and all cation-π interactions can be regarded as occurring on the molecular surface, consistent with earlier suggestions [27]. The overall prevalence of potential cation-π interactions prompts us to suggest that, to some extent, they compete with the classical ion pairs. Thus, the aromatic side chains act as additional transient interaction partners for nearby positively charged side chains, essentially forming a part of the dynamically reorganizing pattern.

In the largely helical structure of the Drebrin-SAH region, all aromatic side chains are largely exposed, the most exposed being Phe16 [180] of the FW motif, leading to the straightforward suggestion that these residues might be important recognition sites for intermolecular interactions (Supplementary Figure S10). However, the cation-π interactions described above might contribute to the partial shielding of aromatic rings, rendering them less exposed to the solvent in the monomeric SAH.

#### Structure, dynamics, and unique features of the Drebrin-SAH region

Simultaneous consideration of global (as revealed by SAXS) and local (as reported by NMR chemical shifts) structural features is indispensable to obtain structural ensembles of SAH regions that reflect their actual dynamics and the extent of their deviations from a straight helical conformation. Apart from the disordered N-terminal residues originating from the expression tag, the dynamical features of the actual SAH region are clearly deducible from both the NMR and SAXS results. Ensemble-based modeling suggests that the Drebrin-SAH forms a stable helical structure with moderate but non-negligible deviations from a fully extended helix. The analysis of side chain interactions reveals a continuous dynamic reorganization of ion pairs and the presence of interactions distinct from the expected i-i+3 and i-i+4 patterns, presumably contributing to the emergence and transient stabilization of non-helical local structures. We propose that the aromatic residues, characteristic of the Drebrin-SAH, take part in this dynamic interaction network by forming various cation-π, and, in the case of the Phe16 [180]-Trp17 [181] pair, aromatic–aromatic interactions. The specific Phe-Trp sequence motif, along with Phe42 [206] and Tyr61 [225], might provide a docking site for potential interacting partners where they can form competing intra- and intermolecular interactions.

The non-uniform structural and dynamical features along the Drebrin-SAH region seem to be dictated by the local sequence, that is, the presence of aromatic residues and the characteristically different Arg/Lys ratio in the N- and C-terminal halves of the helix. This view is supported by the highly conserved nature of the SAH in Drebrin orthologs (Supplementary Table S10), which is somewhat surprising in such a low-complexity region where the major direction of evolutionary pressure could be expected to maintain the overall helical structure. The high conservation of the aromatic residues, together with their location on one side of the helix, reinforces their role as potential interaction sites and also their participation in specific stabilizing interactions.

Compared with the thoroughly characterized myosin 6 and myosin VIIa SAHs, the abundance of aromatic residues is a quite unique feature in Drebrin-SAH. The myosin 6 SAH does not contain a natural aromatic residue, whereas the myosin VII SAH contains mainly histidines beside a single Tyr. Histidine residues can participate in multiple different interactions with positively charged Arg side chains [28], of which the H-bonding interaction is calculated to be the strongest one. In the available myosin VIIa SAH X-ray structure (5WST), only His897 is in the central region of the helix, and it does not interact with any nearby positively charged residue, although its potential interaction with Arg900 cannot be excluded. The other two aromatic residues, His and Tyr, have no assigned coordinates in the construct but are located very close to the termini, unlikely to contribute to the stabilization of the helix.

A survey of SAHs in UniProt suggests that although aromatic residues are not uncommon in predicted SAH domains, and even consecutive aromatic residues can occur, the FW motif is specific for the SAH in Drebrin and the related Drebrin-like protein (Uniprot ID for the human ortholog: Q9UJU6). This suggests that the presence of aromatic side chains is not only compatible with the stable single-helical structure but might be an important functional feature. Further investigation of SAH sequences with different amino acid composition is needed to uncover the exact role of these residues.

## Materials and methods

### Cloning, protein expression, and purification

The DNA sequence of the full Drebrin protein (from *Homo sapiens*, Uniprot Q16643) was a gift from Phillip Gordon-Weeks (Addgene plasmid #40362; http://n2t.net/addgene:40362; RRID: Addgene_40362) and used as template for cloning residues 173–238 into EcoRI and BamHI sites of a modified pET-15b vector (Novagen) with N-terminal 6xHis-tag and TEV cleavage site (ENLYFQG) using primers 5′-ttt ttg gat cca atg AAG CGG ATT AAC CGA GAG CA-3′ (SAH forward) 3′-ttt ttg aat tct aTT TCC TCC TGT GCT CCT CGA TC-5′ (SAH reverse). Expression of the corresponding construct was done with BL21 (DE3) *E. coli* bacteria cells in M9 minimal medium [29]. To produce the necessary isotopically labelled samples, ^15^NH_4_Cl and ^13^C D-glucose were supplemented as the only nitrogen and carbon source. Expression was induced with 1 mM of IPTG (Sigma) at 6 MFU density, and cells were harvested by centrifugation after 3 hours incubation at 37°C. The proteins were extracted from the bacterial pellets with sonication in native phosphate buffer (pH = 7.4, 300 mM NaCl, 43 mM Na_2_HPO_4_, 7 mM NaH_2_PO_4_). The resulting lysate was centrifuged at 14881 × g for 20 min at 4°C, and the supernatant was then filtered through a 0.45-µm filter before purification. The His-tagged protein was purified with a Bio-Scale^TM^ 5 ml Nuvia^TM^ IMAC Ni-affinity column (Bio-Rad). Nonspecifically bound proteins were washed from the column with binding buffer supplemented with 25 mM of imidazole. SAH protein was eluted with an imidazole gradient/250 mM imidazole. TEV protease was used for cleaving the His-tag by incubating with the protein for 20 hours at room temperature. This yielded an eight residue-long (GSHMKDPM) linker remaining upstream the N-terminal of the SAH sequence. Before the ion exchange chromatography, the buffer was changed to a low-salt-binding buffer (20 mM NaCl, 42 mM NaH_2_PO_4_, 8 mM Na_2_HPO_4_, pH = 6.0) using HiTrap Desalting column with Sephadex G-25 resin (Cytiva). For the ion exchange chromatography, Bio-Scale^TM^ Mini Macro-Prep^TM^ High S column was used, and the protein was eluted in a high-salt elution buffer (1 M NaCl, 46 mM Na_2_HPO_4_, 4 mM NaH_2_PO_4_, pH = 7.4). Ultrafiltration was applied using Amicon^®^ Ultra Centrifugal Filter (3 kDa MWCO, Merck, cat#: UFC9003) to concentrate the samples before SEC with SEC70 10 × 300 mm analytical gel chromatographic column (Bio-Rad). The SAH protein construct used for all structural investigations had the following sequence: gshmkdpm KRINREQFWE QAKKEEELRKE EERKKALDER LRFEQERMEQ ERQEQEERER RYREREQQIE EHRRK. The expected MW of the protein assuming natural isotopic abundance is 9727.75 Da. The purity and integrity of the sample was analyzed by SDS-PAGE.

### CD experiments

ECD measurements were conducted at the Separation Science Research and Education Laboratory of Eötvös Loránd University on a Jasco 1500 CD spectrometer (JASCO Corporation, Tokyo, Japan) (A040661638) using a Jasco J/21 cuvette with 1 mm path length. Drebrin-SAH samples were diluted to 14 µM in low-salt buffer (42 mM NaH_2_PO_4_, 8 mM Na_2_HPO_4_, 20 mM NaCl, pH = 6). ECD spectra were recorded at 20°C with the following parameters: 195–250 nm spectral range, 50 nm/min scanning speed, 1 nm bandwidth, 0.2 nm step size, 0.5 s response time, and 3 scans of accumulation. Temperature dependence of helicity was investigated by measuring the ellipticity at 222 nm while heating with a rate of 1°C/min in the temperature range 5–80°C recording with 0.5°C increments. The obtained results were compared with CD data of other proteins, published earlier [15] and kindly provided by Dániel Süveges. Secondary structure content was determined from the mean residual ellipticity values with the BeStSel software [30].

### Backbone assignment experiments

NMR spectra were recorded on an 800 MHz Bruker Avance III HD spectrometer equipped with a ^1^H, ^13^C, ^15^N triple resonance helium-cooled cryoprobe. Initially, the feasibility of ^15^N, ^13^C labeled SAH for structural characterization was evaluated at pH = 7.4 using ^1^H-^15^N HSQC spectra. However, due to severe exchange broadening of several amide cross-peaks, we decided to lower pH to 6.0 in order to reduce the NH exchange rate with the solvent. All further NMR experiments were conducted at 25°C using 350 µM sample in 17 mM NaH_2_PO_4_, 3 mM Na_2_HPO_4_, 50 mM NaCl, pH 6 with 1 mM NaN_3_. Neither the applied change in the pH nor the alteration of the salt concentration is expected to substantially influence the helicity of the construct. The pKa values of the side chains are well outside of the pH range used, and the helicity of other SAHs only decreases at salt concentrations that are higher by about two orders of magnitude [15]. The ^1^H-^15^N HSQC spectrum of Drebrin-SAH only resolved around 50 backbone amide peaks of the total 73 expected, and the standard triple resonance experiments, namely HNCO, i(HCA)CO(CA)NH (also referred as iHNCO), HNCOCACB, HNCACB, resulted in severe signal overlap, as was expected by the repetitive low-complexity nature of the protein sequence. Therefore, the 4D i(HCa)N(Ca)CONH experiment was pivotal to find CO(i)-N(i)-N(i + 1)-HN(i + 1) correlations and lead the sequential walkthrough, supported by the 3D (H)CC(CO)NH experiments to identify residue types by aliphatic C and H chemical shifts. To reduce the long experimental time of the 4D measurement, 7% non-uniform sampling was used with acquisition times of 14.5 ms (^1^H, t_1_), 17.3 ms (^15^N, t_2_), 17.3 ms (^15^N, t_3_), 8 ms (^13^C, t_4_). The ^1^H-^15^N HSQC spectrum was repeatedly measured during the collection of spectra for the assignment to monitor the sample integrity. We observed small signs of degradation after several days; however, the backbone assignment was feasible. All data were processed using Topspin 3.5 software package (RRID:SCR_014227), and assignments were made by CCPN software [31]. Figures have been created by either CCPN or Poky [32]. A list of NMR experiments and their detailed descriptions are available in the supplementary material (Supplementary Table S2).

### SAXS experiments

SAXS data (*I*(*s*) vs. *s*, where *s* = 4psin*q*/l, l is the X-ray wavelength 0.123982 nm (10 keV) and 2*q* the scattering angle) were measured at the EMBL-P12 bioSAXS beam line (DESY, Hamburg Germany [33]) from samples of purified Drebrin-SAH at 88, 175, and 350 µM and a corresponding matched buffer (17 mM NaH_2_PO_4_, 3 mM Na_2_HPO_4_, 50 mM NaCl, pH = 6). Samples were delivered using standard temperature-controlled batch mode operations at 20°C. The data were recorded as sequential sets of 0.1 s individual 2D data frames (Pilatus 6M detector) that underwent subsequent azimuthal averaging to produce 1D scattering profiles normalized to beam transmission [34]. Those 1D data frames assessed to be statistically dissimilar [35], e.g., affected by radiation damage, were removed prior to the final averaging of the sample and buffer frames. The buffer scattering was then subtracted from the sample scattering to produce the final reduced and background-corrected SAXS profiles of the SAH samples at each respective concentration. Additional details on data acquisition and processing may be found in the supplementary material (Supplementary Table S3).

Primary SAXS data analysis was performed using the ATSAS 3 software package [36]. The *Rg* and forward scattering intensity at zero angle, *I(0*), were determined from both the Guinier approximation (ln*(I(s))* vs. *s^2^*, for *sR_g_*< 1.2 [37]) and from the scattering pair distance distribution function, or *p(r*) profile, calculated using GNOM [38] that also provided estimates of the particle maximum dimension, *D*_max_. The Porod volume, *V*_p_, and concentration-independent MV estimates were determined using the ATSAS 3 modules DATPORD and DATMW [39], respectively, using the GNOM outputs for the evaluation. Dimensionless Kratky plots of the SAXS data (*sR_g_^2^(I(s)/I(0*))vs. *sR_g_*) followed the procedure of Receveur–Bréchot and Durand [40]. Additional parameters and details, such as the measured and working *s*-ranges of each dataset, Guinier limits/datapoints used for analysis, quality of parameter fits, and so on, are reported in the Supplementary Material (Supplementary Table S3).

### *Ab initio* dummy atom modeling

The SAXS data from the 350 µM sample underwent *ab initio* dummy atom (DAM) bead modeling to assess the overall structural disposition of SAH. DAMMIF [41] was run several times to generate a 10-member individual DAM model cohort. This cohort underwent subsequent spatial alignment using DAMSEL, DAMSUP, and DAMAVER routines [42] to produce shape, volume, and bead-occupancy-weighted DAM models DAMAVER, DAMFILT, and DAMSTART. The DAMSTART model underwent further refinement in DAMMIN [43] to generate an individual model that fits the data reflecting the overall consensus shape and disposition of SAH2 in solution. In all cases, data-model fits were assessed using the reduced *χ^2^* test and Correlation Map (CorMap) *P-*value [35].

### SEC-MALLS materials and methods

SEC coupled to MALLS and RI measurements were performed to evaluate the molecular mass of the Drebrin-SAH (Supplementary Figure S5). A sample of Drebrin-SAH, 100 ml at 220 µM, was injected onto a Cytiva Superdex Increase 75 5/150 column equilibrated with 17 mM NaH_2_PO_4_, 3 mM Na_2_HPO_4_, 50 mM NaCl, pH = 6 at a flow rate of 0.3 ml/min. MALLS/RI data from the continuously flowing SEC-column eluate were measured using a Wyatt (Germany) miniDAWN^®^ TREOS^®^ (3-angle MALLS) instrument and an Optilab T-rEX refractometer. The RI increment at 25°C for the Drebrin-SAH (dn/dc = 0.1931 ml/g) was estimated using the vbar-dndc calculator of SEDFIT [44] based on the absolute RI of the SEC running buffer (1.3317), the SAH amino acid sequence, incident λ (658 nm), and sample temperature. The subsequent MV estimates through the major SEC elution peak were determined from the MALLS scattering intensities and the RI estimate of protein concentration using Wyatt ASTRA 7.0.1 software.

### Ultra-high-performance liquid chromatography and MALLS size exclusion chromatography experiments

For measuring exact molecular mass, Drebrin-SAH samples in low-salt buffer (42 mM NaH_2_PO_4_, 8 mM Na_2_HPO, 20 mM NaCl, pH = 6, 550 µM) were diluted 50× in H2O and analyzed by Q-TOF MS analysis. To exclude the possibility of potential oligomer forms flying apart and leading to false MW results, additional MALS-SEC experiments were conducted to prove the monomeric state of the protein in solution.

### Conformer generation and selection

Conformer generation was performed with DIPEND [5]. DIPEND uses neighbor-dependent Ramachandran preferences that can be modified by adding a user-defined distribution. Based on the results of several exploratory calculations, we have increased the frequency of sampling helical conformations in several regions of the construct as shown in Supplementary Figure S6. The generated structures were allowed to deviate from a straight helical one, even by allowing the random presence of cis amide bonds not exceeding the frequency observed for non-proline residues [45]. Using these settings, a set of 5000 conformers were generated, each of which was then subjected to a short, 10 ps MD run in vacuum using GROMACS [20] and the AMER99SB-ILDN force field [46], resulting in a total of 30,000 conformers, used as an initial pool. From this initial pool, 5000 conformers were selected randomly, and these were used as an input for the CoNSensX^+^ method to select a subset of conformers corresponding to Cα, Cβ chemical shifts, as well as to the measured SAXS curve. For this purpose, the SAXS evaluation feature was added to the dockerized version of CoNSEnsX^+^ [47] (by incorporating the Pepsi-SAXS method [48]) For the chemical shift selection, the RMSD metric was used, and the SAXS fit was evaluated using the standard *χ*^2^ metric. This procedure – selecting 5000 conformers randomly and then getting a subset with best correspondence to experimental data – was repeated five times.

To assess the role of side chain conformations, backbone-restrained MD calculations of 20 ns were run in explicit water on each conformer that was included in any of the selected ensembles from the initial pool. The AMBER99SB-ILDN force field and the SPC/E water model [49] were used, and structures were put in a rectangular box with walls 1 nm from the nearest atom. Using the default charges, no additional neutralization was necessary. After minimization and two 1-ns equilibration rounds under NVT and NPT conditions with position restraints on all heavy atoms, position restraints on the backbone heavy atoms (N, C, O, Cα) were applied in the production runs. A second pool of conformers was compiled by including 41 conformers from each of the 20-ns MD runs by sampling the structures at every 500 ps. Based on an exploratory analysis, conformations containing cis amide bonds were not included in this pool as they were not required to obtain ensembles with satisfactory correspondence to the experimental (primarily SAXS) data. Termed secondary pool, this conformer set was used as input for a final round of selection based on experimental data with CoNSEnsX^+^. The selection procedure is summarized in Supplementary Figure S7.

Correspondence of the selected ensembles with experimental data was assessed using SHIFTX [50] and Pepsi-SAXS incorporated into CoNSEnsX^+^. In addition, the *Rg* and the distance between the Cα atoms of the terminal residues (Gly1 and Lys74) were calculated for each conformer by in-house Python scripts. Superposition of the structures was performed using MOLMOL [51]. Structural details were visualized with Chimera [52]. Secondary structure analysis was performed using DSSPcont that assigns a probability of each secondary structure state to each residue [53]. DSSPcont output for individual conformers was processed by an in-house script to obtain averaged secondary structure states over the ensemble. The obtained secondary structure state percentages for each residue were used to create an ‘alignment’ file, which could be used as input to WebLogo [54] to generate a logo reflecting the occurrence of secondary structure states for each residue in the ensemble.

The analysis of salt bridges was done with an in-house Perl script according to the criterion described by Barlow and Thornton [55], which is also used in a more recent comprehensive analysis [56]. This requires a distance below 0.4 nm between any side chain oxygen and nitrogen atoms in Asp/Glu and Arg/Lys residues, respectively. Identification of possible cation-π interactions was performed with an in-house script using five characteristic distances between the charged groups of Lys and Arg and the aromatic rings.

Although energy-based identification is favored in the analysis of cation-π interactions [27], in this study, we applied a simple geometry-based criterion for three reasons. First, the analyzed geometries stem from classical MD calculations, providing geometries not expected to precisely match one optimized at a higher level of theory. Second, as also emphasized elsewhere, our model ensemble is not expected to provide a thermodynamically balanced sample and is also too small to represent exhaustive sampling. Thus, the observed interactions can only be regarded as ones that can be reasonably formed during the internal dynamic rearrangements of the side chains, not as contacts supported by direct experimental evidence. Third, we have also used a simple geometry-based criterion for salt bridge identification, and applying similar criteria is more in line with our analysis. For each Phe/Tyr/Trp–Arg/Lys pair, five distances were defined and all of them were required to be below 0.6 nm: (1) the plane of the aromatic ring to Arg Cζ / Lys Nζ , (2) the plane f the aromatic ring to Arg Nε / Lys Cε, (3) Phe/Tyr/Trp Cδ1 to Arg Cζ / Lys Nζ , (4) Phe/Tyr Cδ2 or Trp Cη2 to Arg Cζ / Lys Nζ, and (5) Phe/Tyr Cζ or Trp Cε2 to Arg Cζ / Lys Nζ. Requiring these distances to be below 6 Å still allows various relative orientations of the charged groups to the aromatic rings while ensuring that multiple atoms are close to each other to suggest an interaction of non-negligible strength.

## Supplementary material

Online supplementary figures

Online supplementary tables

## Data Availability

Nuclear magnetic resonance data for the structural ensemble reported in this article have been deposited at the Biological Magnetic Resonance Data Bank, under deposition ID:52729. The corresponding small-angle X-ray scattering data have been deposited to Small Angle Scattering Biological Data Bank with the ID SASDVV6. The generated structural ensemble is available in the Protein Ensemble Database under deposition ID: PED00524.The authors declare that the data supporting the findings of this study are available within the paper and its Supplementary Information files. Should any raw data files be needed in another format they are available from the corresponding author upon reasonable request. The modified version of CoNSEnsX^+^ is available at: https://github.com/PPKE-Bioinf/consensx.itk.ppke.hu (source code) and at https://hub.docker.com/ repository/docker/ppkebioinf/consensx/general (dockerized version).

## Abbreviations

CD: circular dichroism
MALLS: multi-angle laser light scattering
RI: refractive index
SAH: single alpha-helix
SAXS: small-angle X-ray scattering
SEC: size exclusion chromatography
