# Supplementary material for: Dynamic interchange of local residue–residue interactions in the largely extended single alpha-helix in Drebrin
Source: Biochem J. 2025 Apr 23;482(8):383–99. doi: 10.1042/BCJ20253036 (PMC12203971; doi:10.1042/BCJ20253036)
Supplement: Online supplementary figures [file BCJ-482-08-BCJ20253036-s001.docx]

**Dynamic Interchange of Local Residue-Residue Interactions in the Largely Extended Single Alpha-Helix in Drebrin**

Soma Varga^1^, Bálint Ferenc Péterfia^1^, Dániel Dudola^1^, Viktor Farkas^2^, Cy M. Jeffries^3^, Perttu Permi^4,5,6^, Zoltán Gáspári^1^*

[1] Soma Varga, Bálint Ferenc Péterfia, Dániel Dudola, Zoltán Gáspári
Pázmány Péter Catholic University
Faculty of Information Technology and Bionics
1083 Budapest, Práter u. 50/A

[2] Viktor Farkas
ELTE Eötvös Loránd University
HUN-REN - ELTE Protein Modeling Research Group
Pázmány Péter Sétány 1/A,1117 Budapest, Hungary

[3] Cy M. Jeffries
European Molecular Biology Laboratory
Hamburg Unit, c/o Deutsches Elektronen-Synchrotron
Notkestraße 85, 22607 Hamburg, Germany

[4] Perttu Permi
University of Jyväskylä
Department of Biological and Environmental Science, Nanoscience Center
P.O. Box 35, FI-40014, Finland

[5] Perttu Permi
University of Jyväskylä
Department of Chemistry, Nanoscience Center
P.O. Box 35, FI-40014, Finland

[6] Perttu Permi
University of Helsinki
Helsinki Life-Science Institute – Institute of Biotechnology
P.O. Box 56, FI-00014, Finland

*Correspondence: Zoltán Gáspári ([zoltan.gaspari@itk.ppke.hu](mailto:zoltan.gaspari@itk.ppke.hu), https://orcid.org/0000-0002-8692-740X )

SUPPLEMENTARY MATERIAL

| 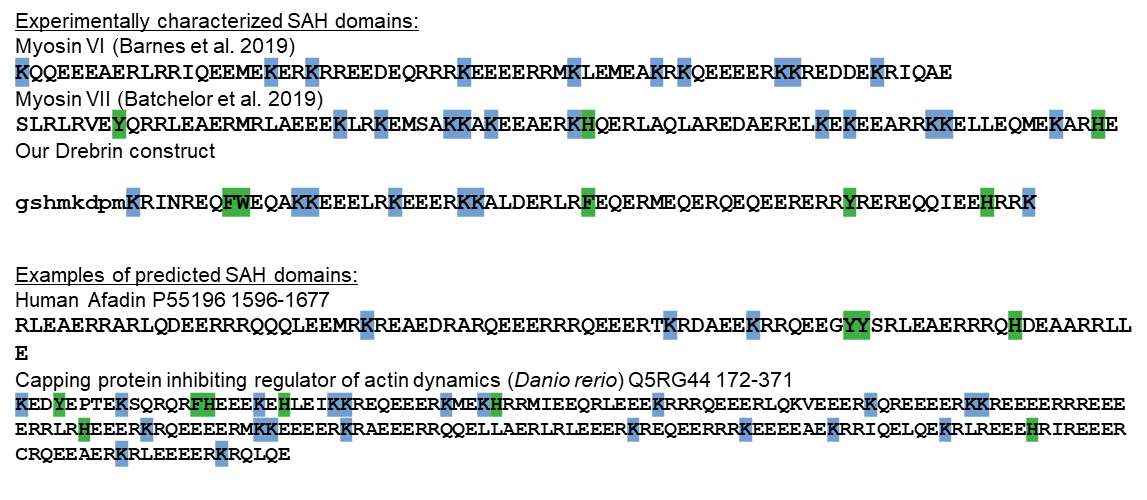  Figure S1. Comparison of the Drebrin SAH region with SAH segments that have been extensively characterized by NMR so far. Lys residues are highlighted with blue background and aromatic residues with green background. Additonal examples of two predicted SAH domains in two actin-interacting proteins. Both contain consecutive aromatic residues and exhibit a different distribution of lysines along the sequence. |
| --- |
| 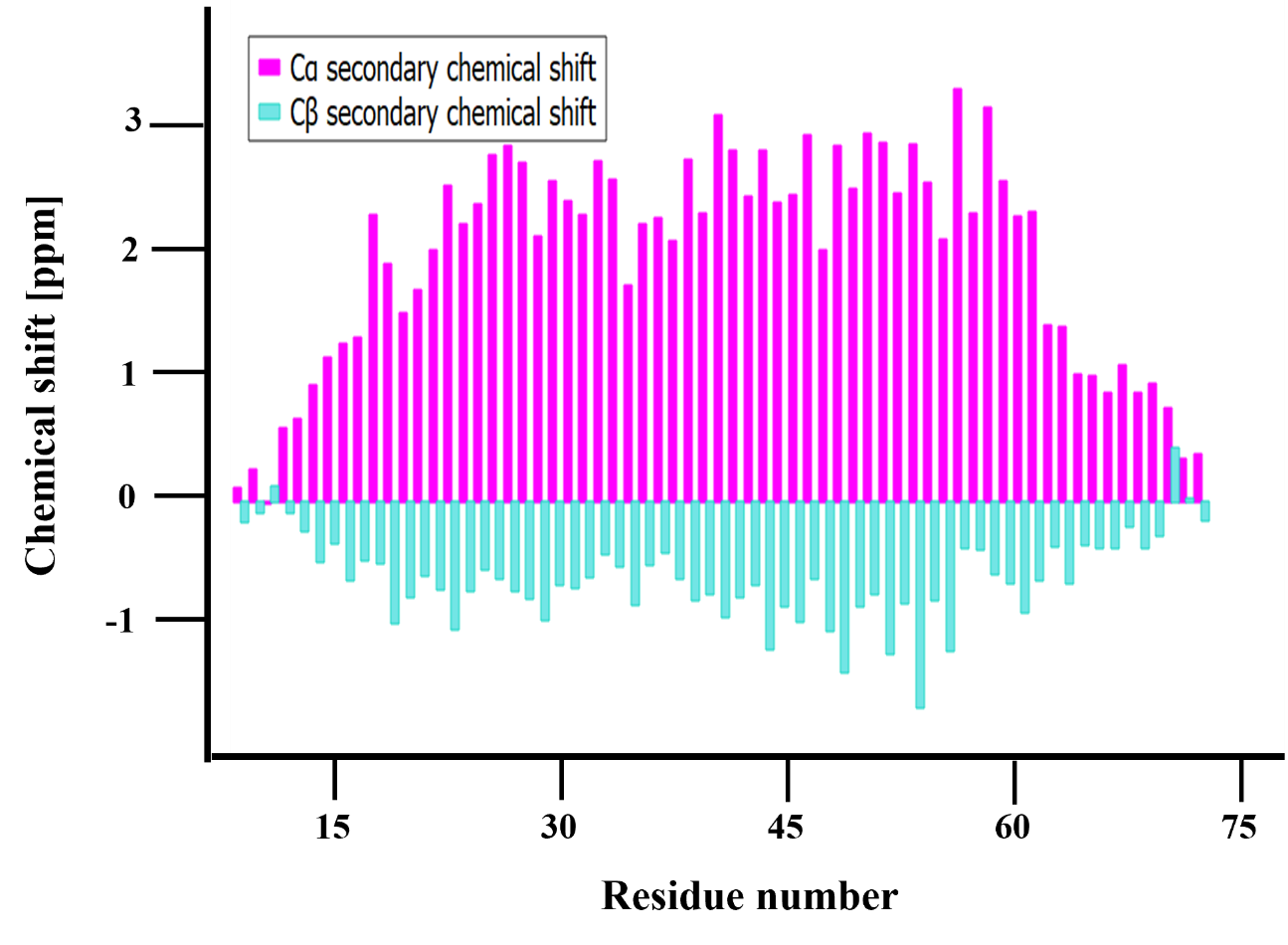 |
| Figure S2. Secondary chemical shifts of Drebrin SAH calculated with POTENCI. Magenta columns: secondary Cα chemical shifts, high positive difference from random coil values indicate the local helicity Teal columns: secondary Cβ chemical shifts, the negative difference from random coil values further indicate the local helicity. |

| 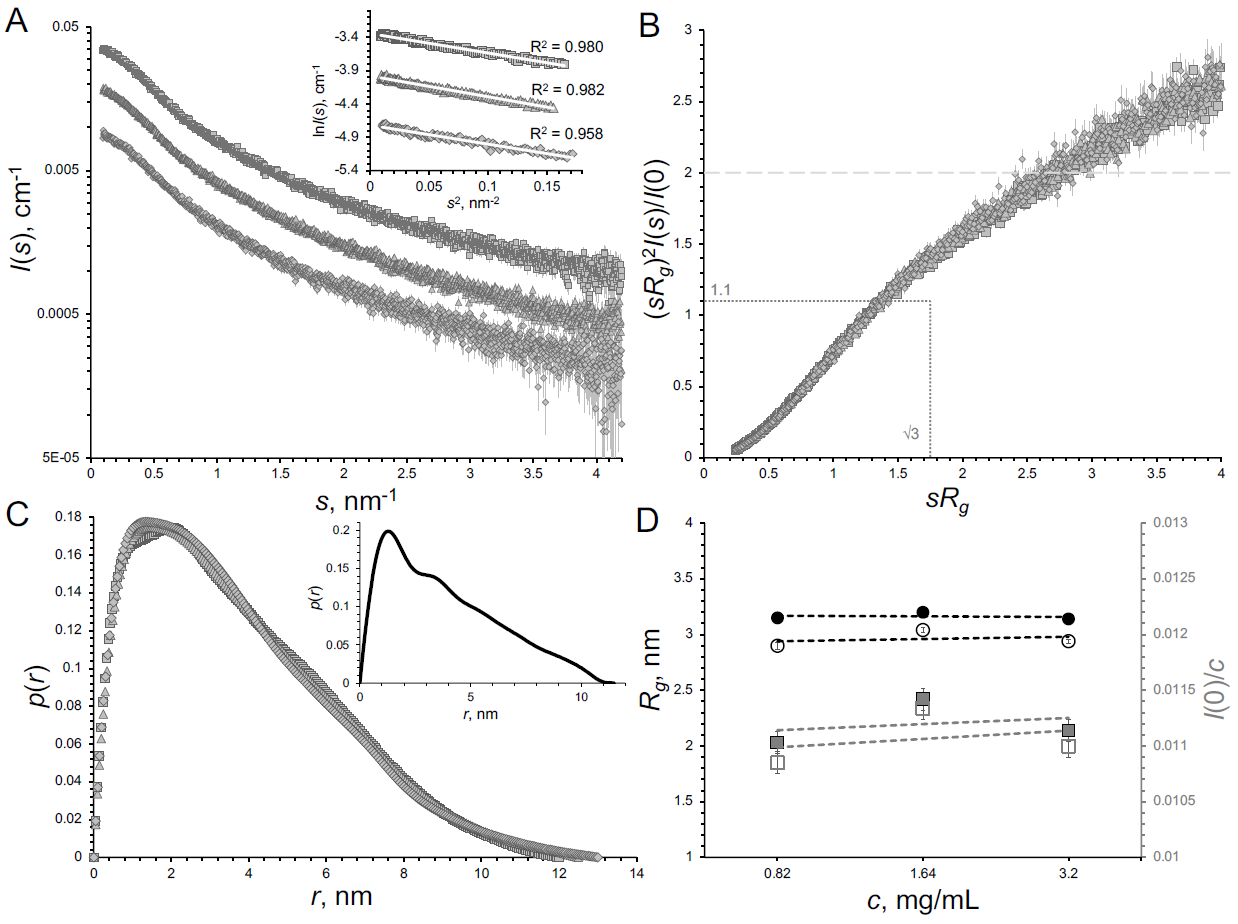 |
| --- |
| **Figure S3: SAXS data and plots for the SAH of Drebrin.** **A.** Background-subtracted SAXS data (working *s*-range) from three sample concentrations of Drebrin. From top to bottom: 3.2 mg/mL (grey squares), 1.64 mg/mL (grey triangles) and 0.84 mg/mL (grey diamonds). *Inset*: Corresponding Guinier plots and respective linear fits to the Guinier region at very-low angles (~0.28 < *sR_g_* < 1.2). (Table S3) **B.** Dimensionless Kratky plots for the three SAXS datasets qualitatively demonstrating the extended nature/states of the SAH population in solution (a ‘globular maximum’ is absent in the transformed scattering intensities at 1.1, √3 and the data do not plateau toward ~2 at higher *sR_g_*, which is otherwise indicative of an intrinsically disordered protein). **C.** Real-space scattering-pair distance distributions (*p*(*r*) profiles, area-normalized to 1) calculated from the SAXS datasets in **A** showing the highly anisotropic distribution of distances that extend to a *D_max_* of 12–13.5 nm. *Inset*: The *p*(*r*) calculated from a single-particle model of drebrin-SAH where the α-helix adopts an extended ‘stiff’ rod-shaped conformation (model 48 of the NMR ensemble). **D.** The dependence of the *R_g_* (black) and *I*(0)/*c* (grey) as a function of protein concentration, *c*, demonstrating little-to-no concentration effects on these structural parameters. Closed circles/squares: parameters obtained from *p*(*r*); Open circles/squares: parameters determined from the Guinier approximation. |


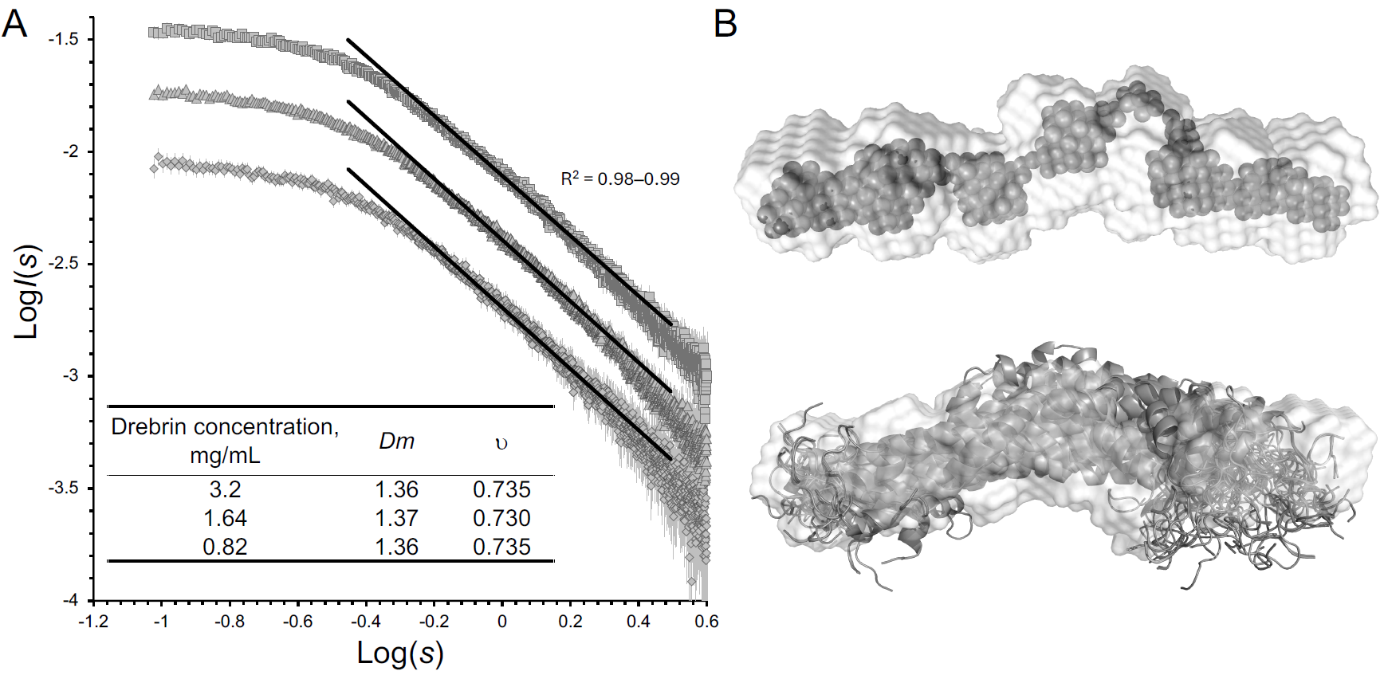


**Figure S4:**  **A** Log-Log scale representation of the SAXS data measured from drebrin-SAH as described in Figure S3 (top to bottom: 3.2 mg/mL (grey squares), 1.64 mg/mL (grey triangles) and 0.84 mg/mL (grey diamonds)), with corresponding estimates of the decay in the scattering intensities determined from the linear slope of Log*I*(*s*) vs Log(*s*) across the mid-*s* region of each profile (~0.55–2.0 nm^-1^; black lines). The mass fractal dimension, *Dm*, and corresponding estimate of the Flory exponent (υ, where υ ~ 1/*Dm*) are consistent with the scaling behaviour of extended, but not stiff/linear rod-like particles. **B.** *Top*: Examples of the shape of SAH reconstructed from the 3.2 mg/mL SAXS data profile showing the anisotropic/extended distribution of mass (volume occupancy) of an individual bead model (grey spheres) aligned into to the envelope generated from the minimized spatial alignment of a 10-model cohort (white surface). *Bottom*: A spatial alignment and comparison between the drebrin-SAH model-ensemble (grey ribbons) derived from NMR and the overall shape of a spatially-aligned dummy-atom 10-model cohort reconstructed from the 3.2 mg/mL SAXS dataset (white surface).


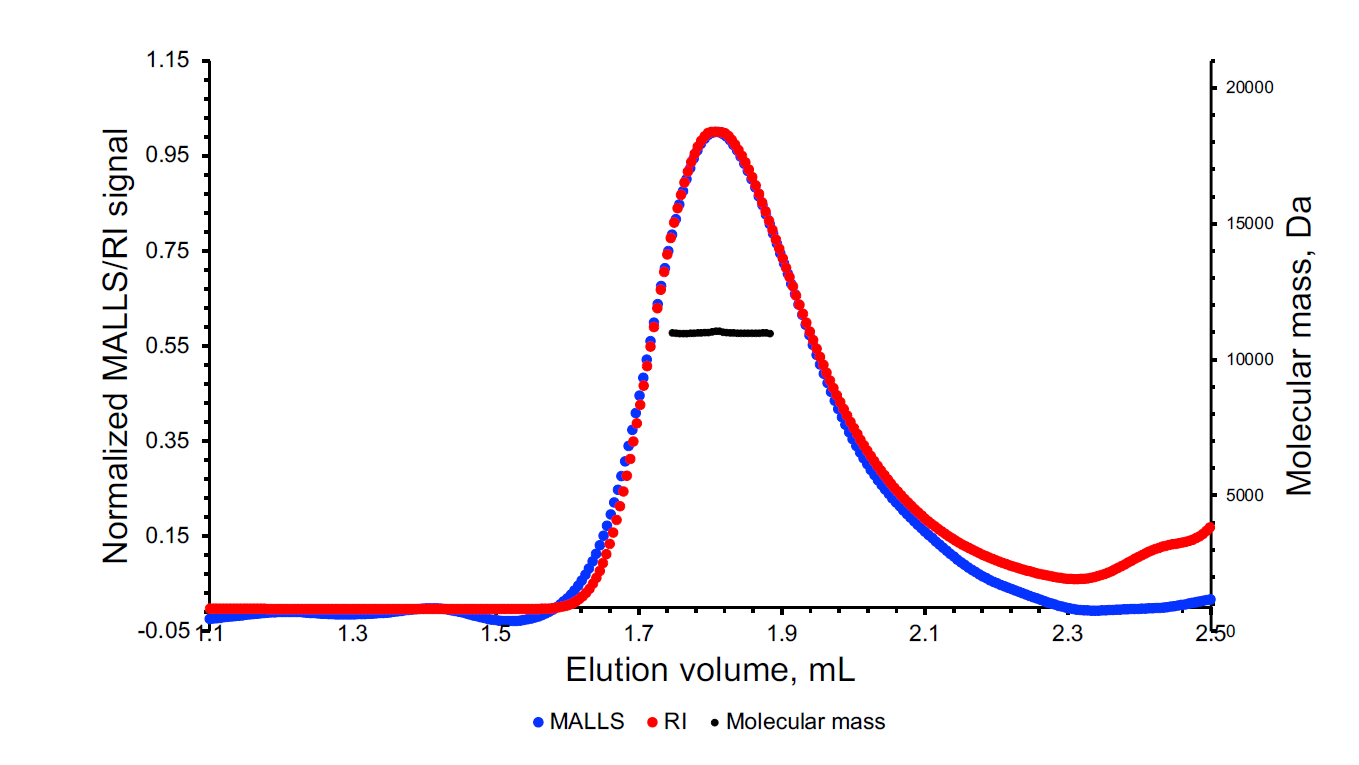


**Figure S5: Size exclusion chromatography (SEC) MALLS/RI analysis of Drebrin-SAH:** Multi-angle laser light scattering trace (MALLS, Rayleigh ratio, blue) and differential refractive index (dRI, red) SEC-elution profiles with the associated calculated experimental molecular mass estimates/correlation through the SEC peak (~11 kDa) demonstrating that the SAH is a monomer in solution (expected molecular mass, monomer = 9.7 kDa).


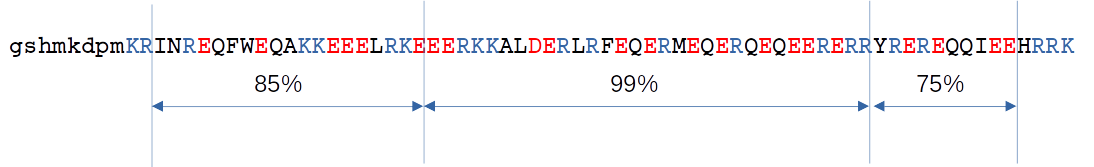


Figure S6. Helical preferences of different regions of the Drebrin SAH construct used for conformer generation with DIPEND.

| 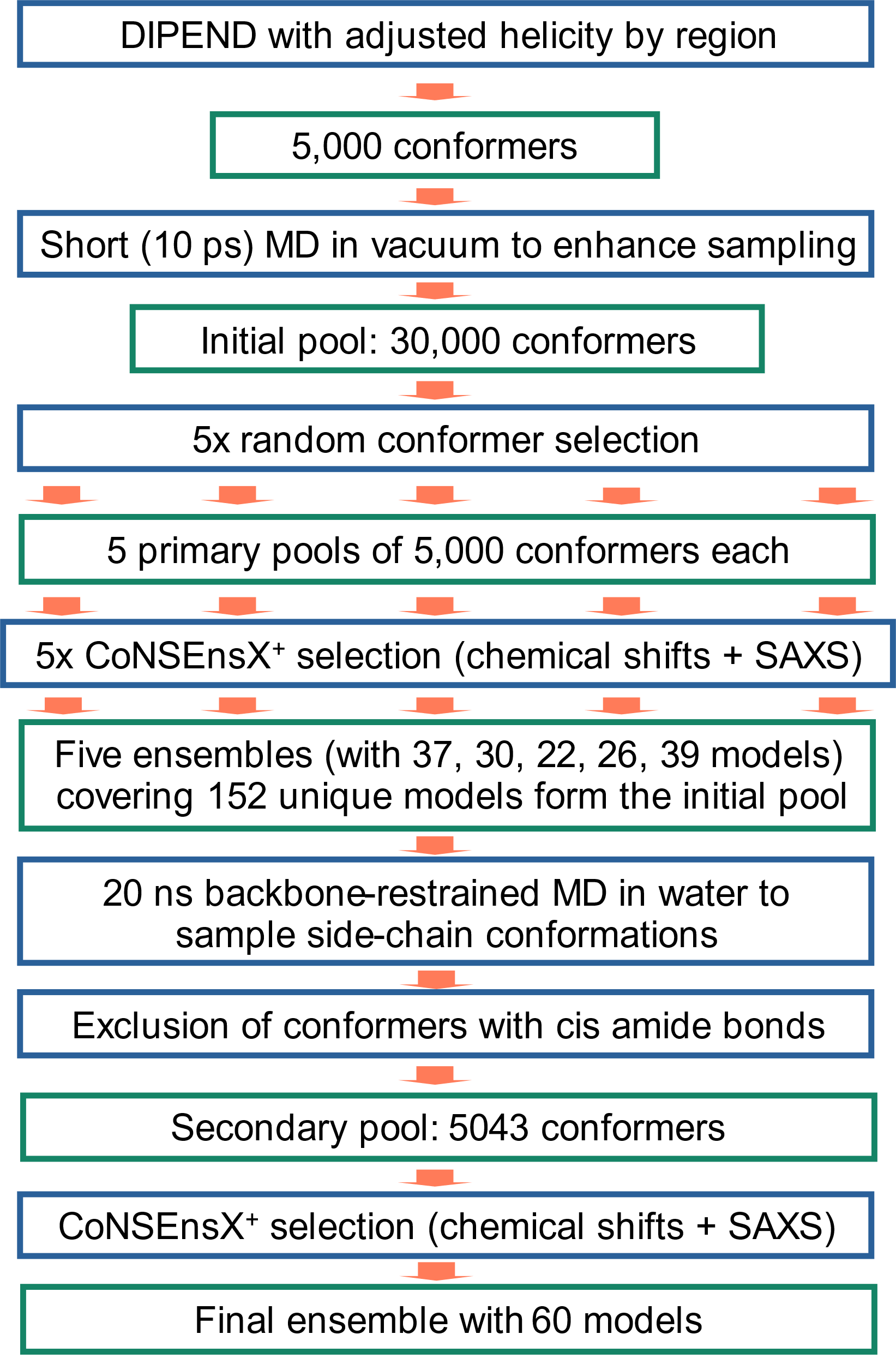 |
| --- |
| Figure S7. Scheme of the ensemble selection protocol applied. |

|  |  |
| --- | --- |
| Figure S8. Principal component analysis of the initial pool with the models selected from the primary pools highlighted. The selected conformers do not sample the full extent of the PC1 and PC3 coordinates, indicating clear structural preferences for the selected ensembles.  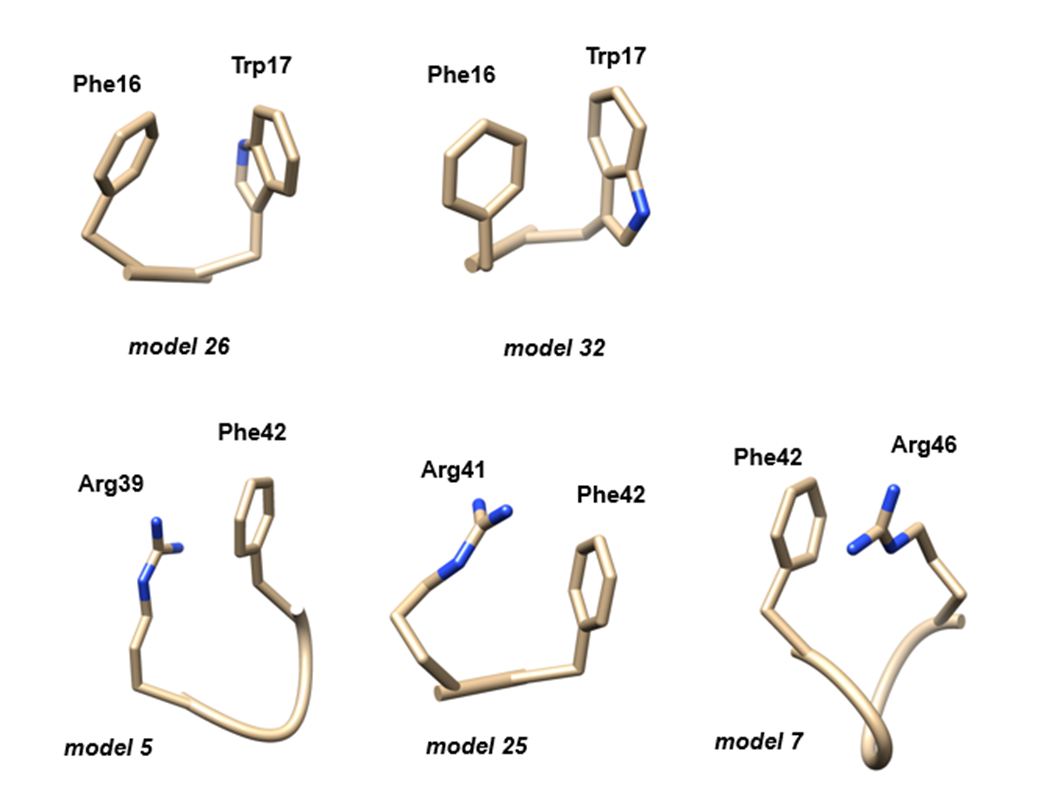  Figure S9. Interactions between the aromatic side chains of Phe16 and Trp17. A parallel arrangement suggesting the presence of a pi:pi interaction, as well as an edge-to-face interaction.  Putative cation:pi interactions formed by Phe42 with Arg39, Arg41 and Arg46.  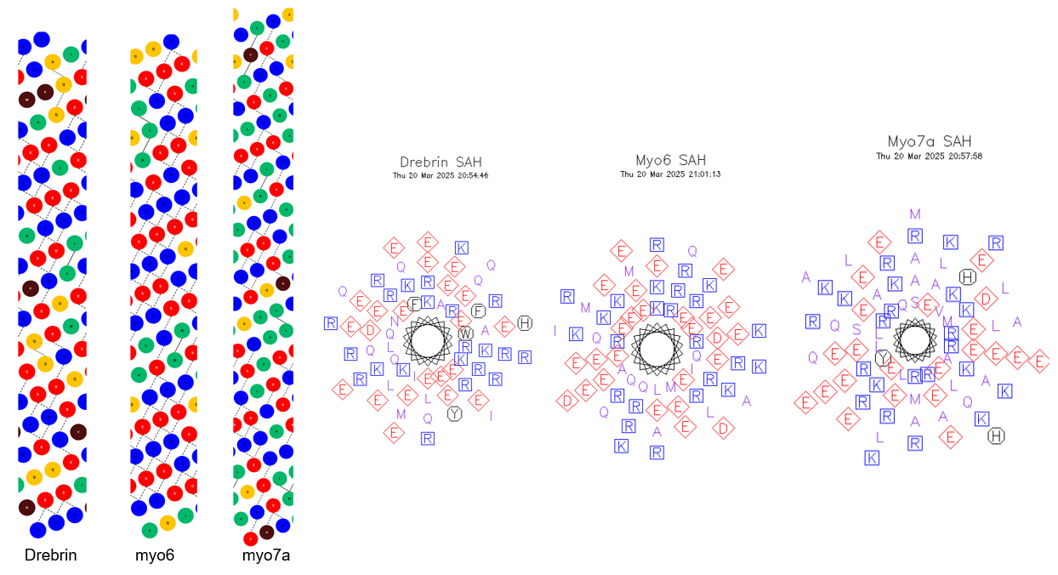 | |

Figure S10. Helical net and helical wheel representations of the Drebrin. Myo6 and Myo7a SAH regions. The helical wheel generated for Drebrin SAH shows that the aromatic residues are located on one face of the helix. Figures were generated with pepwheel (https://www.bioinformatics.nl/cgi-bin/emboss/pepwheel) and NetWheel (18) (http://lbqp.unb.br/NetWheels/).

Table S1.

| Reference | Protein | Residue nr | Positive | Positive % | Negative | Negative% | Charged % |
| --- | --- | --- | --- | --- | --- | --- | --- |
| Barnes 2019 | Myo6 | 68 | 25 | 37 | 26 | 38 | 75 |
| Batchelor 2019 | Myo7a | 80 | 27 | 34 | 22 | 28 | 61 |
| this study | Drebrin | 66 | 23 | 35 | 22 | 33 | 68 |

Table S2: List of NMR experiments used for resonance assignment

| **Experiment** | **Reference** | **Used for** |
| --- | --- | --- |
| ^1^H-^15^N HSQC with Rance-Kay SE, water flip-back pulse ^15^N | ^1^ | Fingerprint of NH residues |
| HNCO with Rance-Kay SE ^15^N, ^13^C | ^2^ | Triple resonance residue i-1 CO assignment |
| iHNCO with Rance-Kay SE ^15^N, ^13^C | ^3^ | Triple resonance residue i CO assignment |
| (H)CC(CO)NH with Rance-Kay SE DIPSI-2 spin-lock ^15^N ^13^C | ^4^ | Residue i-1 aliphatic side chain ^13^C assignment |
| H(CC)(CO)NH with Rance-Kay SE DIPSI-2 spin-lock ^15^N ^13^C | ^4^ | Residue i-1 aliphatic side chain ^1^H assignment |
| (HACA)N(CA)CONH with Rance-Kay SE ^15^N ^13^C | ^5^ | 4D CO(i)-N(i)-(Ni+1)-HN(i+1) correlations |

Table S3: **SAXS Reporting Table****:** Extended single alpha helix (SAH) of Drebrin.

| Sample details | | | |  |
| --- | --- | --- | --- | --- |
| Organism | *Homo sapiens* | | |  |
| *Scattering particle composition* |  |  |  |  |
| Protein | **Drebrin** – UniProt [Q16643](https://www.uniprot.org/uniprotkb/Q16643/entry) amino acids 172–238 single alpha helix incorporating an additional eight non-native amino acids at the N-terminus (GSHMKDPM) derived from the remnants of a protein affinity tag, post TEV cleavage. | | |  |
| *Sample environment* |  |  |  |  |
| Solvent composition | 17 mM NaH_2_PO_4_, 3 mM Na_2_HPO_4_, 50 mM NaCl, pH 6 | | |  |
| Sample temperature | 20 °C | | |  |
| In beam sample cell | 1 mm pathlength in-vacuum quartz capillary; continuous flow | | |  |
| Sample concentration, *c* | 0.82 mg/mL | 1.64 mg/mL | 3.2 mg/mL |  |
| SAS data collection | | | |  |
| Data acquisition/reduction software | EMBL bioSAXS *SASFLOW* pipeline^6^ ; *BECQUEREL* beam line control software^7^ . Batch-mode robot sample delivery. | | |  |
| Source/instrument description or reference | EMBL P12 bioSAXS beam line^8^ equipped with a Pilatus 6M detector (Dectris). Sample-to-detector distance = 3 m. *s*-axis calibrated to silver behenate. | | |  |
| X-ray wavelength (energy) | λ = 0.123982 nm (10 keV) | | |  |
|  | SAH, 0.82 mg/mL | SAH, 1.64 mg/mL | SAH, 3.2 mg/mL |  |
| Measured *s*-range (*s_min_*–*s_max_* nm^-1^) | 0.023–4.43 | | |  |
| Working *s*-range (*s_min_*–*s_max_* nm^-1^)* | 0.095–4.00 | 0.095–4.07 | 0.086–4.27 |  |
| Method for scaling intensities | Absolute scaling relative to the scattering from water, cm^-1^ and normalized to beam transmission | | |  |
| Exposure time (number of sample frames used for averaging) | 0.1 s (101) | 0.1 s (77) | 0.1 s (20) |  |
| SAS-derived structural parameters | | | |  |
| Methods/Software | *PRIMUS* (ATSAS 3.0)^9^ including *GNOM 5*^10^ | | |  |
| *Guinier Analysis* | SAH, 0.82 mg/mL | SAH, 1.64 mg/mL | SAH, 3.2 mg/mL |  |
| *I*(0)/*c* ± σ (mL.cm^-1^.mg^-1^) | 0.01085 (0.0001) | 0.01134 (0.0001) | 0.01100 (0.0001) |  |
| *R*_g_ ± σ (nm) | 2.90 (0.03) | 3.04 (0.02) | 2.94 (0.02) |  |
| *min < sR_g_* < *max* limit (data point range) † | 0.29–1.20 (28–137) | 0.29–1.20 (26–130) | 0.28–1.20 (26–134) |  |
| Linear fit assessment (Pearson correlation coefficient^2^, R^2^) | 0.958 | 0.982 | 0.980 |  |
| *P(*r*) analysis* |  |  |  |  |
| *I*(0)/*c* ± σ (mL.cm^-1^.mg^-1^) | 0.01103 (0.0001) | 0.01142 (0.0001) | 0.01114 (0.0001) |  |
| *R*_g_  ± σ (nm) | 3.15 (0.05) | 3.20 (0.02) | 3.14 (0.02) |  |
| *d*_max_ (nm) | 13.0 | 12.5 | 12.0 |  |
| *s*-range (nm^-1^) | 0.10–4.0 | 0.124–4.04 | 0.095–4.18 |  |
| *P*(*r*) fit assessment *χ*^2^ (CorMap-*P*) | 0.98 (0.485) | 1.06 (0.486) | 1.01 (0.293) |  |
| Scattering particle size | | | |  |
| Methods/Software | ATSAS 3: *DATPOROD*, *DATMW*^11^ modules | | |  |
| *Volume estimates* | SAH, 0.82 mg/mL | SAH, 1.64 mg/mL | SAH, 3.2 mg/mL |  |
| Porod volume, *V_p_* (nm^3^)^a^ | 17.8 | 18.8 | 17.9 |  |
| *Molecular weight estimates (kDa)* |  |  |  |  |
| Expected MW calculated from amino acid sequence | 9.73 | | |  |
| MW from SAXS/*P*(*r*): DatBayes^a^ (MW range; > 0.9 confidence) | 8.5 (6.6–16.5) | 8.5 (6.6–9.1) | 8.5 (6.6–9.1) |  |
| *From SAS-independent method*: MALLS/RI (± σ) | - | - | 10.985 (0.024) |  |
| MW ratio (MALLS/expected) | - | - | 1.13 |  |
| RI dn/dc value (*SEDFIT*)^12^ |  |  | 0.1931 mL/g |  |
| *Ab initio* bead modelling | | | |  |
| Methods/Software | Dummy-atom shape/volume reconstruction using *DAMMIF*^13^*/DAMMIN*^14^ | | |  |
| *Dataset used for shape reconstruction* | SAH, 3.2 mg/mL, *GNOM 5* input. | | |  |
| *s-*range for individual model building (*s_min_*–*s_max_* nm^-1^) | 0.095–4.18 |  |  |  |
| Symmetry | P1 |  |  |  |
| # of individual model reconstructions | 10 *DAMMIF* models, fast-mode, spatially aligned with *DAMSEL*, *DAMSUP*, *DAMAVER*^15^ (normalized spatial discrepancy across the model cohort = 0.939). DAMSTART output used as input into *DAMMIN*. | | |  |
| Model fit *χ*^2^ (CorMap-*P*) | 0.992 (0.259) (evaluated across the *s*-range 0.095–3.64 nm^-1^) | | |  |
| Data and model deposition SASBDB accession code: SASDVV6 | | | |  |

*The working *s*-range was determined using a combination of *AUTORG*^16^ (*s*_min_) and *SHANUM*^17^ (*s*_max_). †Point range refers to the data points of the measured SAXS profiles of each dataset (not the working *s*-range); refer to the SASBDB entry SASDVV6 for the SAXS profiles measured across the full *s*-range at each sample concentration. ^a^Porod volume and concentration-independent MW estimates from the SAXS data were calculated using the corresponding GNOM.out files as input into *DATPOROD* and *DATMW*, respectively.

Tables S4-S10 are provided in a separate Excel file:

Table S4. Correspondence of different ensembles to selected experimental data

Table S5. Experimental and calculated chemical shifts for the final ensemble

Table S6. Radius of gyration and end-to-end distance of the models in the final ensemble

Table S7. Averaged DSSPCont results for the final ensemble

Table S8. Atom-atom distances indicating the presence of salt bridges in the final ensemble

Table S9. Distances indicating the presence of cation-π interactions in the final ensemble

Table S10.Sequences producing significant alignments for NCBI BLASTP on the Drebrin SAH sequence

References:

(1) Iii, A. P., and Wright, P. Sensitivity Improvement in Proton-Detected Two-Dimensional Heteronuclear Correlation NMR Spectroscopy. *JOURNAL OF MAGNETIC RESONANCE*. DOI: 10.1016/j.jmr.2014.05.006

(2) Grzesiek, S., and Bax, A. D. (1992) Improved 3D Triple-Resonance NMR Techniques Applied to a 31 kDa Protein. *JOURNAL OF MAGNETIC RESONANCE*. DOI: 10.1021/bi00082a001

(3) Mäntylahti, S., Tossavainen, H., Hellman, M., and Permi, P. (2009) An intraresidual i(HCA)CO(CA)NH experiment for the assignment of main-chain resonances in 15N, 13C labeled proteins. *J Biomol NMR* *45*, 301–310. DOI: 10.1007/s10858-009-9373-4

(4) Montelione, G. T., Lyons, B. A., Emerson, S. D., and Tashiro, M. (1992) An efficient triple resonance experiment using carbon-13 isotropic mixing for determining sequence-specific resonance assignments of isotopically-enriched proteins. *J Am Chem Soc* *114*, 10974–10975. DOI:

10.1021/ja00053a051

(5) Tossavainen, H., Salovaara, S., Hellman, M., Ihalin, R., and Permi, P. (2020) Dispersion from Cα or NH: 4D experiments for backbone resonance assignment of intrinsically disordered proteins. *J Biomol NMR* *74*, 147–159. DOI: 10.1007/s10858-020-00299-w

(6) Franke, D., Kikhney, A. G., and Svergun, D. I. (2012) Automated acquisition and analysis of small angle X-ray scattering data. *Nucl Instrum Methods Phys Res A* *689*, 52–59. https://doi.org/10.1016/j.nima.2012.06.008

(7) Hajizadeh, N. R., Franke, D., and Svergun, D. I. (2018) Integrated beamline control and data acquisition for small-angle X-ray scattering at the P12 BioSAXS beamline at PETRAIII storage ring DESY. *J Synchrotron Radiat* *25*, 906–914. DOI: 10.1107/S1600577518005398

(8) Blanchet, C. E., Spilotros, A., Schwemmer, F., Graewert, M. A., Kikhney, A., Jeffries, C. M., Franke, D., Mark, D., Zengerle, R., Cipriani, F., Fiedler, S., Roessle, M., and Svergun, D. I. (2015) Versatile sample environments and automation for biological solution X-ray scattering experiments at the P12 beamline (PETRA III, DESY). *J Appl Crystallogr* *48*, 431–443. DOI: 10.1107/S160057671500254X

(9) Manalastas-Cantos, K., Konarev, P. V., Hajizadeh, N. R., Kikhney, A. G., Petoukhov, M. V., Molodenskiy, D. S., Panjkovich, A., Mertens, H. D. T., Gruzinov, A., Borges, C., Jeffries, C. M., Svergun, D. I., and Franke, D. (2021) *ATSAS 3.0* : expanded functionality and new tools for small-angle scattering data analysis. *J Appl Crystallogr* *54*, 343–355. DOI: 10.1107/S1600576720013412

(10) Svergun, D. I. (1992) Determination of the regularization parameter in indirect-transform methods using perceptual criteria. *J Appl Crystallogr* *25*, 495–503.

(11) Hajizadeh, N. R., Franke, D., Jeffries, C. M., and Svergun, D. I. (2018) Consensus Bayesian assessment of protein molecular mass from solution X-ray scattering data. *Sci Rep* *8*, 7204. DOI: 10.1038/s41598-018-25355-2

(12) Schuck, P. (2000) Size-Distribution Analysis of Macromolecules by Sedimentation Velocity Ultracentrifugation and Lamm Equation Modeling. *Biophys J* *78*, 1606–1619. DOI: 10.1016/S0006-3495(00)76713-0

(13) Franke, D., and Svergun, D. I. (2009) *DAMMIF* , a program for rapid *ab-initio* shape determination in small-angle scattering. *J Appl Crystallogr* *42*, 342–346. DOI: 10.1107/S0021889809000338

(14) Svergun, D. I. (1999) Restoring Low Resolution Structure of Biological Macromolecules from Solution Scattering Using Simulated Annealing. *Biophys J* *76*, 2879–2886. DOI: 10.1016/S0006-3495(99)77443-6

(15) Volkov, V. V., and Svergun, D. I. (2003) Uniqueness of *ab initio* shape determination in small-angle scattering. *J Appl Crystallogr* *36*, 860–864. https://doi.org/10.1107/S0021889803000268

(16) Petoukhov, M. V., Konarev, P. V., Kikhney, A. G., and Svergun, D. I. (2007) *ATSAS* 2.1 – towards automated and web-supported small-angle scattering data analysis. *J Appl Crystallogr* *40*, s223–s228.

https://doi.org/10.1107/S0021889807002853

(17) Konarev, P. V., and Svergun, D. I. (2015) *A posteriori* determination of the useful data range for small-angle scattering experiments on dilute monodisperse systems. *IUCrJ* *2*, 352–360. DOI: 10.1107/S2052252515005163

(18) Mól A.R., Castro M.S. and FonteS W. (2024) NetWheels: A Web Application to Create High Quality Peptide Helical Wheel and Net Projections. *Journal of Bioinformatics and Systems Biology. 7*: 98-100.
